# Towards Effective Non-Invasive Brain-Computer Interfaces Dedicated to Gait Rehabilitation Systems

**DOI:** 10.3390/brainsci4010001

**Published:** 2013-12-31

**Authors:** Thierry Castermans, Matthieu Duvinage, Guy Cheron, Thierry Dutoit

**Affiliations:** 1 TCTS lab, Université de Mons, Place du Parc 20, Mons 7000, Belgium; E-Mails: matthieu.duvinage@gmail.com (M.D.); Thierry.Dutoit@umons.ac.be (T.D.); 2 LNMB lab, Université Libre de Bruxelles, Avenue Franklin Roosevelt 50, Bruxelles 1050, Belgium; E-Mail: gcheron@ulb.ac.be

**Keywords:** brain-computer interface, brain dynamics, brain imaging, electroencephalography, rehabilitation, supra-spinal control of locomotion, walk

## Abstract

In the last few years, significant progress has been made in the field of walk rehabilitation. Motor cortex signals in bipedal monkeys have been interpreted to predict walk kinematics. Epidural electrical stimulation in rats and in one young paraplegic has been realized to partially restore motor control after spinal cord injury. However, these experimental trials are far from being applicable to all patients suffering from motor impairments. Therefore, it is thought that more simple rehabilitation systems are desirable in the meanwhile. The goal of this review is to describe and summarize the progress made in the development of non-invasive brain-computer interfaces dedicated to motor rehabilitation systems. In the first part, the main principles of human locomotion control are presented. The paper then focuses on the mechanisms of supra-spinal centers active during gait, including results from electroencephalography, functional brain imaging technologies [near-infrared spectroscopy (NIRS), functional magnetic resonance imaging (fMRI), positron-emission tomography (PET), single-photon emission-computed tomography (SPECT)] and invasive studies. The first brain-computer interface (BCI) applications to gait rehabilitation are then presented, with a discussion about the different strategies developed in the field. The challenges to raise for future systems are identified and discussed. Finally, we present some proposals to address these challenges, in order to contribute to the improvement of BCI for gait rehabilitation.

## 1. Introduction

More than 10 million people in the world live with some form of handicap caused by a central nervous system (CNS) disorder. According to a recent Eurostat survey carried out in 25 European countries, about 15% of the active population suffer from a long-term disability This means that almost 45 million persons of working age, *i.e.*, 15 to 64, live with such a medical condition. Disabilities affecting mobility, in particular, often lead to exacerbated isolation and, thus, fewer communication opportunities, resulting in a limited participation in social life. Encounters with other people are made difficult, as well as simply performing usual daily tasks at home and, obviously, working. Lower limb disability can have various origins, either medical (after a stroke, multiple sclerosis or Parkinson's disease, for instance) or accidental (road traffic accident, sport practice accident, *etc.*). In these conditions, either leg muscles become inefficient for walking or the brain motor signals do not even properly reach the spinal motoneurons commanding the leg muscles. The consequences are similar: the disabled person cannot properly stand up or walk anymore. Developing technological tools to empower the lower limbs of disabled people with walking ability will drastically change their day-to-day life, as they will perform most usual daily activities more independently, both at home and outside, thus sustaining their own inclusion in society.

In 2009, it was shown that invasive recordings of ensembles of cortical neurons in primary motor and primary somatosensory cortices could be used to predict the kinematics of bipedal walking in rhesus macaques [1]. The same year, another research team demonstrated that specific combinations of serotonergic agonists and epidural electrical stimulation were able to restore the motor control of adult rats after complete spinal cord transection [2]. More recently, epidural stimulations of the spinal cord enabled a young paraplegic patient to achieve full weight-bearing standing, with assistance provided only for balance, for more than 4 min. This technique also allowed him to produce locomotor-like patterns and control some leg movements [3]. These recent breakthroughs are highly encouraging for developing revolutionary rehabilitation strategies. However, the generalization of this type of clinical rehabilitation is far from being applicable to all patients suffering from spinal cord injuries or other CNS movement disorders.

The major challenge for walking rehabilitation in human arises from the fact that since the very first step, the human CNS must dynamically integrate both conservative (postural stability) and destabilizing (dynamic control of the body and limbs for forward progression) functions [4,5,6,7]. These two antagonistic functions render the rehabilitation of human locomotion very challenging. This also explains why the majority of leg prostheses available on the market are equipped with passive mechanisms. Although these systems are functional, their performance is really limited compared to a real human leg, as they do not have a self-propulsion capability. Therefore, amputees have to compensate for these limitations, and they are generally faced with a reduced locomotion speed, a non-natural gait, considerable fatigue and, possibly, harmful consequences, like recurrent pain and injuries at the interface between their residual limb and the prosthesis. Active prostheses solve these problems partially: powered by a battery-operated motor, they move on their own and, therefore, reduce the fatigue of the amputees, while improving their posture. Two main categories of active prostheses exist to date. Firstly, by analyzing the motion of the healthy leg or the upper-body by means of sensors, the control system can identify the phase of the gait cycle and trigger an actuator to appropriately adjust one or more prosthetic or orthotic joint [8,9,10,11,12]. The second type of active prostheses (or orthoses) is controlled by myoelectric signals recorded at the surface of the skin, just above the muscles. These signals are then used to guide the movement of the artificial limb [13,14,15].

Although the improvement brought by active prosthetic technology with respect to conventional prostheses is indisputable, an intuitive interface from which users intent can be determined is still missing. The purpose of this paper is to review, firstly, the substantial progress made in the understanding of human locomotion control and, in the second part, the exploitation of this knowledge that is being made in order to develop non-invasive brain-computer interfaces dedicated to walk rehabilitation systems. Section 2 summarizes the main mechanisms involved in human locomotion control. Section 3 focuses on the description of supra-spinal control of locomotion by summarizing the knowledge acquired to date thanks to multiple methods of measuring neuronal activity. Section 4 discusses different strategies developed to produce walk rehabilitation systems driven by non-invasive brain-computer interfaces.

## 2. Deciphering Human Locomotion Control

Accumulating evidence suggests that human locomotion is actually based on a very complex hierarchical system, which includes several control networks located both at the spinal and supra-spinal levels. Basically, high-level motor commands are sent by the brain to a spinal network composed of central pattern generators (CPGs), and at the same time, each level of motor control receives and transmits peripheral sensory information (sensory feedback), which is used to modify the motor output at that level. This section is first devoted to the description of each level of locomotor control, including arguments supporting the existence of a CPG network and, simultaneously, the permanent action of supra-spinal control. Then, the focus is on the spatial organization of supra-spinal control and its temporal characteristics.

### 2.1. Description of the Gait Cycle

**Figure 1.**
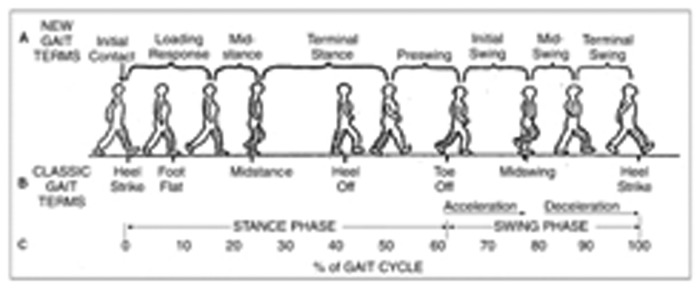
Illustration of different phases of the gait cycle. (**A**) New gait terms; (**B**) classic gait terms; and (**C**) Percentage of gait cycle. Note: this figure is adapted with permission from [16]; Copyright Demos Medical Publishing Inc., 2004.

Human walking is composed of successive periodic and symmetric movements produced by a precise sequence of collective actions, one leg alternating with the other one. The gait cycle is usually defined as starting with the first contact (initial contact, or heel contact in normal gait) of one foot, so that the end of the cycle occurs with the next contact of the same (ipsilateral) foot (see Figure 1). Each cycle begins with a stance phase (when the foot hits the ground) and proceeds through a swing phase, until the cycle ends with the limb's next initial contact.

**Figure 2:**
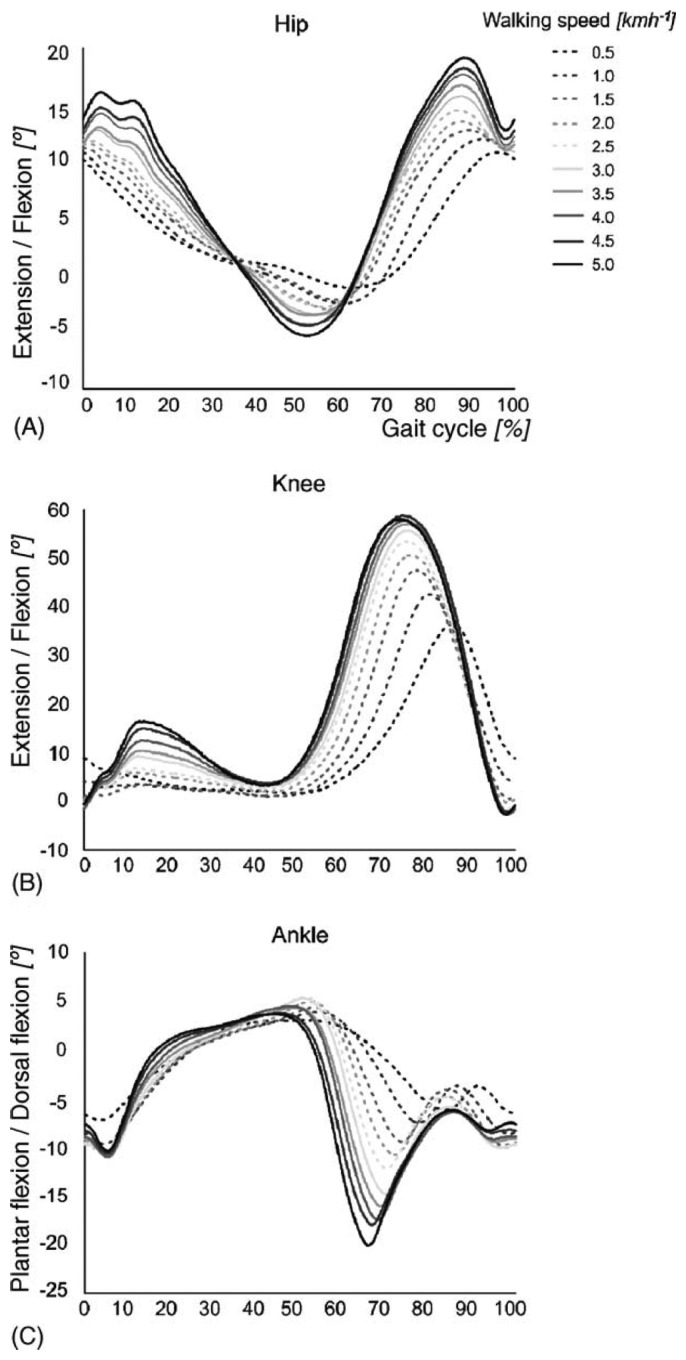
Influence of walking speed on joint trajectories. Joint trajectories of the (**A**) hip, (**B**) knee and (**C**) ankle joint at 10 different walking speeds (this figure is reproduced with permission from [17]; Copyright IOS Press, 2010).

The stance phase of gait is divided into four periods: loading response, mid-stance, terminal stance and preswing. The swing phase is divided into three periods: initial swing, mid-swing and terminal swing. The beginning and ending of each period are defined by specific events, listed in Table 1. Figure 2 presents the typical joint kinematics of the lower body during the gait cycle, for a range of walking speeds [17]. Although the amplitude of the hip joint movement clearly increases as a function of the walking speed, the movement pattern remains about the same, except at very slow walking speeds. A similar behavior is found for the knee joint. Clear changes in amplitude and movement pattern occur in the ankle joint, already at speeds slower than 3.0 km/h.

**Table 1:** Subdivisions of the stance and swing phases of the gait cycle.

**Phase**		**Events**
**Stance**	Loading response (0%–10%)	Begins with initial contact, the instant when the foot contacts the ground. Normally, the heel contacts the ground first. The loading response ends with the contralateral toe off, when the opposite extremity leaves the ground. Thus, the loading response corresponds to the gait cycle's first period of double limb support.
	Mid-stance (10%–30%)	Begins with the contralateral toe off and ends when the center of gravity is directly over the reference foot. Note that this phase, and early terminal stance, the phase discussed next, are the only times in the gait cycle when the body's center of gravity truly lies over the base of support.
	Terminal stance (30%–50%)	Begins when the center of gravity is over the supporting foot and ends when the contralateral foot contacts the ground. During terminal stance, the heel rises from the ground.
	Preswing (50%–60%)	Begins at the contralateral initial contact and ends at the toe off. Thus, the preswing corresponds to the gait cycle's second period of double limb support.
**Swing**	Initial swing (60%–70%)	Begins at toe off and continues until maximum knee flexion (60 degrees).
	Mid-swing (70%–80%)	The period from maximum knee flexion until the tibia is vertical or perpendicular to the ground.
	Terminal swing (80%–100%)	Begins where the tibia is vertical and ends at initial contact.

The stance phase lasts approximately 60% of the gait cycle, while the swing phase occurs during the remaining 40% of the time. Each gait cycle includes two periods of double support, when both feet are in contact with the ground. The first double support begins at initial contact and lasts for the first 10% to 12% of the cycle. The second period of double limb support occurs in the final 10% to 12% of the stance phase.

### 2.2. Production of the Basic Locomotor Patterns: Arguments in Favor of a Human CPG Network

**Figure 3:**
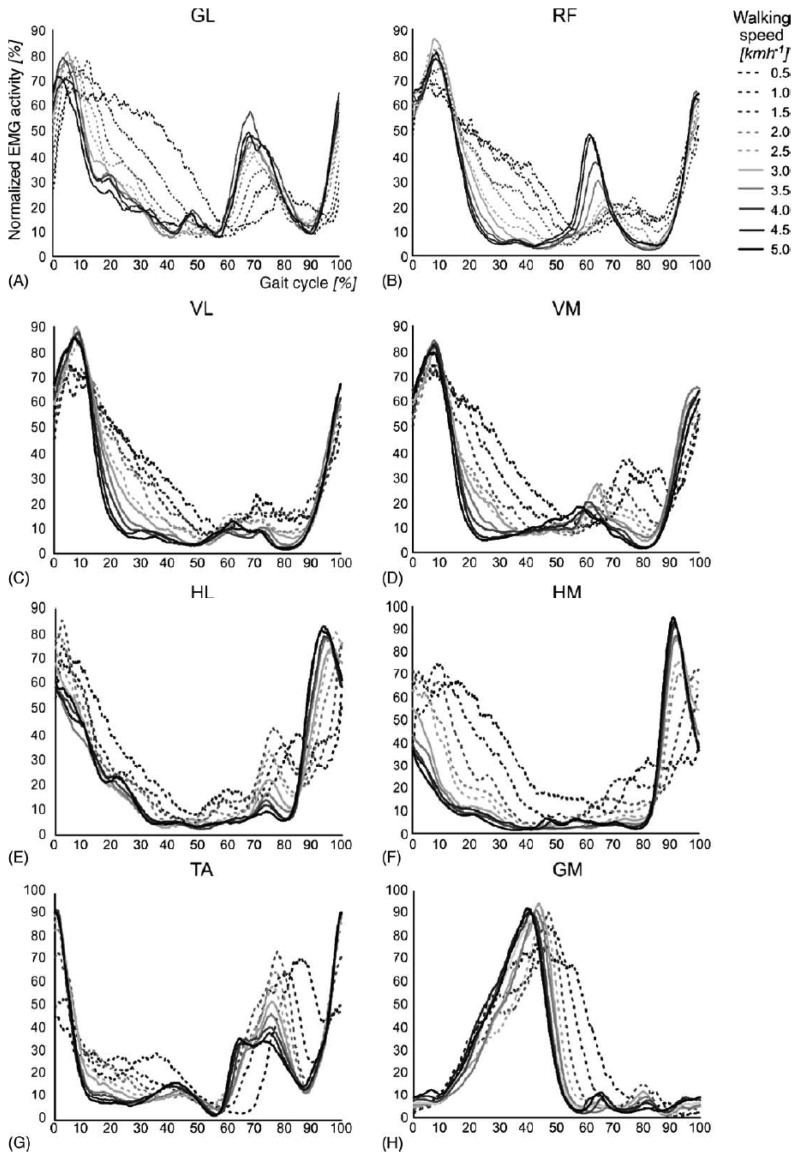
Influence of walking speed on electromyographic (EMG) activity patterns. EMG activity patterns during different walking speeds of the (**A**) gluteus maximus (GL); (**B**) rectus femoris (RF); (**C**) vastus lateralis (VL); (**D**) vastus medialis (VM); (**E**) lateral hamstrings (HL); (**F**) medial hamstrings (HM); (**G**) tibialis anterior (TA); and (**H**) gastrocnemius medialis (GM) muscles. EMG signals were normalized for each subject and each condition by setting the difference between the lowest and highest EMG amplitude at 100% and normalizing the curve according to this value (this figure is reproduced with permission from [17]; Copyright IOS Press, 2010).

The rhythmic movements of the legs during stepping require a complex sequence of muscle contractions to be executed by the lower limbs. The timing and level of activity of the numerous muscles involved differ widely, as illustrated in Figure 3. The complex sequence of muscle contractions is called the motor pattern for stepping. These patterns vary as a function of walking speed, the largest differences occurring between 2.0 and 2.5 km/h for most muscles. A growing body of evidence suggests that the patterns for stepping in mammals are produced at the spinal level by the central pattern generators network [18].

The spinal central pattern generator network consists of coupled antagonist oscillators specifically dedicated to extensor or flexor muscles acting at the different joints. This network generates the rhythm and shapes the pattern of the motor bursts of motoneurons [19,20]. Their mechanism allows one to produce simple and coordinated rhythmic movements, such as those involved in steady walking. Numerous experiments with spinal cats (*i.e.*, with complete transection of the spinal cord) have demonstrated the presence of such CPG in lower mammals [20], and a similar conclusion has been reached for primates [6]. Regarding humans, the evidence is only indirect [21,22].

The first argument is the fact that human infants exhibit a stepping behavior from birth [23] and even before birth, as seen from ultrasound [24] or imaging recordings [25], although the brain has a weak influence on the lower limbs movements at this early stage of development. Similar patterns are seen in a variety of other immature mammals [26,27,28].

Furthermore, studies with young infants stepping on a split-belt treadmill have shown that the stepping patterns in the two legs could be independent, but always remained coordinated (only one leg entering the swing phase at a time), resulting in an integer relationship between the steps on each side. This finding is in favor of the CPG hypothesis, because a stepping movement that would be due to a reflex mechanism (e.g., such as the stretch reflex, a muscle contraction in response to stretching in the muscle) would not exhibit such precise coordination. Other studies with babies have also shown that their stepping mechanism responds to different perturbations the same way as the one of lower mammals, for which the existence of CPG is almost certain [29]. Recently, this analogy has been verified on the basis of experimental results consistent with the hypothesis that, despite substantial phylogenetic distances and morphological differences, locomotion in several animal species is built starting from common primitives, perhaps related to a common ancestral neural network [30].

Another argument supporting the theory of a human CPG comes from patients exhibiting involuntary rhythmic spontaneous leg movements after both clinically complete [31,32] and incomplete [33] spinal cord injury (SCI), thus with minimal influence of cortical signals. Similarly, sleep-related periodic leg movements have been reported. These stereotyped, periodic, repetitive movements involve one or both lower limbs. They consist of dorsiflexion of the ankle and toes and flexion of the hip and knee while the subject is lying down or asleep [34,35]. The spinal origin of such movements is supported by their presence in patients with complete spinal lesion.

A final piece of evidence that CPG is at the basis of our rhythmic locomotor activity and can be located in the spinal cord comes from experiments in which specific sites of the spinal cord were electrically stimulated. Indeed, it was shown that tonic electrical stimulation of the dorsal side of the spinal cord could induce locomotor activity in intact, decerebrated and low spinalized cats [36,37,38,39]. A similar spinal cord stimulation applied to persons with a complete spinal lesion eliciting a stepping activity with reciprocal, organized EMG activity of symmetric muscles [21]. This suggests that a comparable neural network (CPG) to that seen in the cat is present in humans.

### 2.3. Sensory Feedback Regulates the Stepping Patterns

Normal walking is generally considered an automatic movement. However, it is not necessarily stereotyped. We constantly use sensory input to adjust stepping patterns to variations of the terrain or to unexpected events. Three different types of sensory information are integrated to regulate our way of stepping: somatosensory input from the receptors of muscles and skin, input from the vestibular apparatus (balance control) and visual input [18].

Sensory feedback, elicited during gait, acts directly on the CPG and plays a major role in the phase transitions during the step cycle [40]. In particular, it was shown that limb loading and hip position are powerful signals for regulating the stepping pattern in human infants [41].

**Figure 4:**
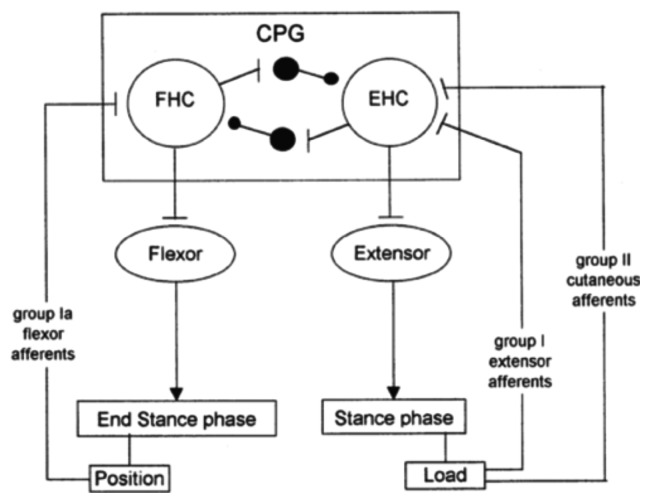
Model of the different pathways indicating how afferents can act on the central pattern generator (CPG) during the stance phase of locomotion. The CPG contains a mutually inhibiting extensor and flexor half-center (EHC and FHC, respectively). During the stance phase, the load of the lower limb is detected by group I extensor muscle afferents and group II (low threshold) cutaneous afferents, which activate the EHC. In this way, extensor activity is reinforced during the loading period of the stance phase. At the end of the stance phase, group Ia afferents of flexor muscles excite the FHC (which inhibits the EHC) and, thereby, initiate the onset of the swing phase (this figure is reproduced with permission from [42]; Copyright Elsevier, 1998).

Cutaneous reflexes are also known to contribute to the correct execution of leg movements during locomotion. They are largely under the control of the CPG. In this way, it is ensured that reflex activations of given muscles occur at the appropriate times in the step cycle and are suppressed at other times [42], as illustrated in Figure 4. This reflex activity, which regulates the timing and amplitude of the stepping patterns [18,43,44], takes place at very specific moments in the gait cycle. In other phases of locomotion, the motor cortex seems to become especially active. In particular, during normal walking, the tibialis anterior (TA) shows two activity periods, one at the end of the stance and one at the end of the swing. It has been suggested that the first burst is primarily due to output of a spinal CPG, whereas the second is more of cortical origin [45]. Indeed, clinical observations on stroke patients clearly show that, especially, the second burst (end swing) is affected after damage to the motor cortex. Additionally, transcranial magnetic stimulation studies during gait have also pointed toward a strong involvement of the motor cortex in the generation of this activity [46].

In other contexts than normal locomotion, sensory input from the skin also allows stepping to adjust to unexpected obstacles [18]. The reflex mechanism, by its own, can give rise to a bipedal locomotion system that is stable, reproduces human walking dynamics and leg kinematics, tolerates ground disturbances and adapts to slopes without parameter interventions, as modeled in [47]. However, arguments exposed hereafter indicate that human locomotion comprises more than a CPG network modulated by reflexes.

### 2.4. Evidence of a Supra-Spinal Control

Although the existence of a CPG system modulated by sensory information has become broadly accepted, many findings indicate that the cortex also plays a role of primary importance in human walking [22]. Indeed, when lesions occur in the supra-spinal region of the central nervous system, recovery of walking is extremely difficult and generally incomplete. This means that intact supra-spinal centers are necessary for functional walking in humans.

Experiments with mammals also provide strong arguments in favor of a supra-spinal control. For instance, after transection of their spinal cord, most cats are not able to generate locomotor movements. This observation suggests that commands for the initiation of locomotor activity must be given at a certain level above the lesion. By varying the level of transection, it was shown that the regions for initiation of locomotion are located in the brain stem, *i.e.*, at the supra-spinal level [48]. Furthermore, in paralyzed decerebrated cats, the initiation of ‘osfictive’ locomotion (*i.e.*, in the absence of movement-related afferent feedback) can be realized with electrical stimulation of the mesencephalic locomotor (MLR) region [49]. Such MLR regions have also been described in different vertebrate species, including primates [50].

Another kind of evidence for supra-spinal control of locomotion is provided by the effects of substances mimicking the action of descending pathways. Many studies have shown that a walking pattern can be elicited in acute spinalized cats put on a treadmill after intravenous injection of such substances [51,52,53]. Furthermore, it is even possible to modify the walking pattern and modulate the step cycle duration and step length by varying the nature of the injected drugs [54,55].

Furthermore, studies of direct stimulation of neurons in the motor cortex through transcranial magnetic stimulation have shown that the motor cortex likely plays a role in activating the dorsiflexors and plantarflexors during walking in humans [56]. Additionally, significant changes in motor and cognitive demands (*i.e.*, spatial attention) have been observed in the context of bipedal walking in unknown or cluttered dynamic environments [57]. Functional neuroimaging studies have shown that the primary motor cortex is recruited during rhythmic foot or leg movements [58]. Moreover, the technique of functional near-infrared spectroscopy (fNIRS) has allowed for the detection of the involvement of the frontal, premotor and supplementary motor areas during walking [59]. Electrophysiological studies have also provided valuable information concerning the possible cortical origin of the intramuscular and intermuscular electromyographic (EMG) synchronization (coherence) observed in lower limbs during walking [60].

Finally, numerous studies have revealed strong arguments supporting the idea that motor centers in the brain play an important and greater role in human walking compared with quadrupeds, as reviewed in [6,61,62].

### 2.5. An Overview of the Human Locomotion Machinery

**Figure 5:**
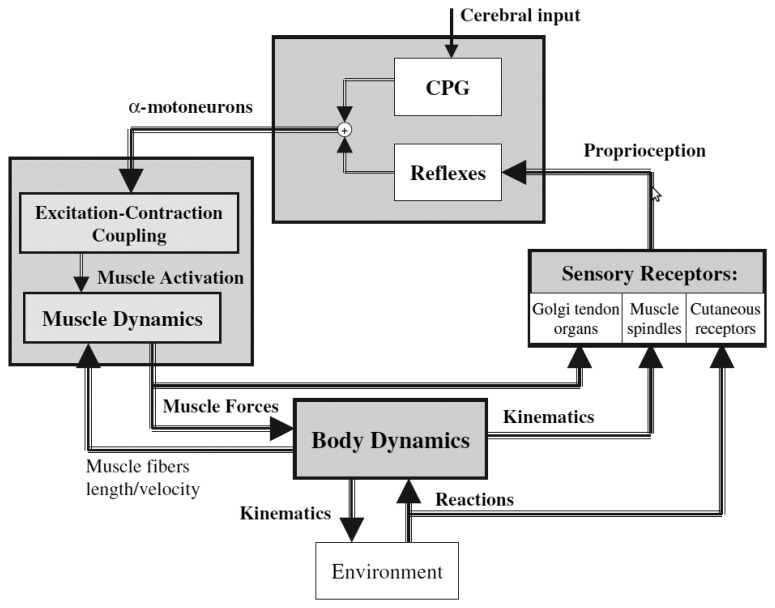
Global view of the human locomotion machinery (this figure is reproduced with permission from [63]; Copyright Springer-Verlag, 2005; see text for details).

Figure 5 gives a global picture of the human locomotion control process and summarizes the different points discussed so far. Initiation of the movements, rhythm modulation and stopping come from the superior central nervous system (*i.e.*, the brain). Brain signals are sent to the spinal cord, where the complex spinal circuitry manages to decode them (along with the feedback afferent signals coming from the peripheral nervous system). The CPG network produces rhythmic patterns of neural signals. The resulting command signals, called efferent impulses, are emitted by the *α* motoneurons and transmitted through the motor nerves to the muscles. By contracting in response to the nervous solicitations, muscles produce active forces, which are transmitted to the skeleton through the tendons. The forces generate the movements of the limbs. The feet interact with the ground, and external forces push the body forward. The balance of the body is ensured by feedback thanks to the proprioceptive organs that respond to mechanical stimuli by generating electrical impulses (action potentials). These action potentials are sent back to the spinal cord through the afferent sensory nerves. The muscle spindles determine the muscle fiber lengths and velocities, while the Golgi tendon organs provide information about the muscle forces. Specific cutaneous mechanoreceptors located in the skin are able to detect tension, changes in texture, rapid vibrations, sustained touch, pressure and stretches. Additional mechanoreceptors are also found in the joints.

All this feedback information is integrated in the spinal cord in order to automatically stabilize the walking, by means of reflexes (*i.e.*, without intervention of the brain). This mechanism is valid for limited perturbations and, in the case of important perturbations, the superior central nervous system and the vestibulo-oculomotor system have to intervene, so as to prevent the fall.

## 3. Supra-Spinal Control of Human Locomotion

In this section, we focus on the role of the brain in human locomotion control. Spatial organization and temporal characteristics of supra-spinal control are described, with an emphasis on the information that can be detected in a non-invasive way. These aspects are particularly important for the discussion conducted in Section 4, which concerns the development of non-invasive brain-computer interface (BCI) dedicated to walk rehabilitation systems.

### 3.1. Measuring Brain Activity

Brain imagery techniques are essential tools to investigate the spatial and temporal organization of the supra-spinal centers involved, for instance, in human locomotion control. To date, these techniques allow one to monitor two types of brain activities: first, the electrophysiological activity and, second, the hemodynamic response of the brain.

The electrophysiological activity of the brain is produced both by the electro-chemical transmitters exchanging information between the neurons and by the ionic currents generated within the neurons themselves. Electrophysiological activity can be measured thanks to electroencephalography (EEG), electrocorticography (ECoG), magnetoencephalography (MEG) and invasive electrical measurements operated at the single neuron level.

The hemodynamic response of the brain allows one to distinguish active from less activated neurons. Indeed, the blood releases glucose to active neurons at a greater rate than in the area of inactive neurons. The presence of glucose and oxygen results in a surplus of oxyhemoglobin in the veins of the active area. Hence, the local ratio of oxyhemoglobin to deoxyhemoglobin changes [64]. The variations of this ratio can be quantified by methods, such as functional magnetic resonance and near-infrared spectroscopy, from which it is possible to build 3D maps of the brain activity These kinds of methods are often considered as indirect, because they measure the hemodynamic response, which, in contrast to electrophysiological activity, does not directly characterize the neuronal activity.

In the following paragraphs, each brain imaging technique is explained. First, electrophysiological methods are discussed, and then, metabolic methods are described. Table 2 summarizes the different imaging techniques by listing in each case the type of brain activity measured, the temporal and spatial resolutions, safety and portability (adapted from [65]).

**Table 2:** An overview of neuroimaging methods. Direct methods detect electrical or magnetic activity of the brain, while metabolic methods are considered as indirect methods of imaging (adapted from [65]). EEG, electroencephalographic; MEG, magnetoencephalography; ECoG, electrocorticography; LFP, local field potential; MUA, multi-unit activity; SUA, single-unit activity; fMRI, functional magnetic resonance imaging; SPECT, single-photon emission-computed tomography; PET, positron-emission-tomography; NIRS, near-infrared spectroscopy.

**Neuroimaging Method**	**Activity Measured**	**Temporal Resolution**	**Spatial Resolution**	**Risk**	**Portability**
EEG	Electrical	∼0.001 s	∼ 10 mm	Non-invasive	Portable
MEG	Magnetic	∼0.05 s	∼ 5 mm	Non-invasive	Non-portable
ECoG	Electrical	∼0.003 s	∼ 1 mm	Slightly invasive	Portable
Intracortical neuron recording	Electrical	∼0.003 s	∼0.5 mm (LFP)	Strongly invasive	Portable
			∼0.1 mm (MUA)		
			∼0.05 mm (SUA)		
fMRI	Metabolic	∼1 s	∼ 1 mm	Non-invasive	Non-portable
SPECT	Metabolic	∼10 s–30 min	∼ 1 cm	Non-invasive	Non-portable
PET	Metabolic	∼0.2 s	∼ 1 mm	Non-invasive	Non-portable
NIRS	Metabolic	∼1 s	∼2 cm	Non-invasive	Portable

#### 3.1.1. Electroencephalography

Electroencephalography (EEG) measures the electric brain activity caused by the currents induced by neurons and during synaptic excitations of the dendrites [66]. The measurements are realized thanks to electrodes placed on the scalp, thus in a non-invasive way. This explains why EEG is by far the most widespread brain activity recording modality. With NIRS (see below), EEG is the only non-invasive acquisition technique that is really portable. Moreover, it is relatively cheap and offers a high temporal resolution (about 1 ms). However, EEG scalp electrodes are only able to measure the electrical potentials of thousands of neurons, which are weakened and smeared by the volume conduction effect of the skull [67], leading to signals of a few microvolts only and a poor global spatial resolution.

The weak amplitude of EEG signals renders them sensitive to electronic noise and artifacts. EEG artifacts are spurious signals present in recordings and whose origin is not cerebral. They may arise from the patient itself: the eyes, the tongue, the pharyngeal muscle, the scalp muscles, the heart or the sweat glands all produce electrical potentials, which can influence the EEG measurement, especially if they are in movement. Skin resistance changes, due to sweating, may also badly affect the signals. Electrical interference with a power line or surrounding electrical apparatus is another source of artifacts that may be induced electrostatically or electromagnetically. Finally, artifacts may also arise from faulty electrodes or the recording equipment itself.

In many cases, artifacts can be immediately identified by visual spatial analysis: high amplitude potentials appearing at only one electrode are not likely due to cerebral activity. Indeed, brain produces potentials that exhibit a physiological distribution characterized by a maximum voltage amplitude gradually decreasing with increasing distance over the scalp. Likewise, rhythmical or repetitive irregular signals appearing simultaneously in non-adjacent brain areas strongly suggest the presence of artifacts [68].

Algorithms designed to detect and correct EEG artifacts integrate these principles and exploit techniques, like temporal filtering, spatial filtering, independent component analysis (ICA) [69,70], blind source search (BSS) [71] or thresholding of meaningful parameters (e.g., channel variance) based on a prior statistical analysis [72].

EEG analysis of human locomotion is particularly complicated by experimental difficulties [73,74]: in addition to “traditional” EEG artifacts (ocular, muscular, power line, *etc.*), EEG recordings realized in ambulatory conditions are further degraded by additional sources of noise. Triboelectric noise is generated by movement, friction and flexion of the cable components, resulting in a static or piezoelectric movement transducer effect [75]. Electrode movements are produced by movements of the head, but also by the shocks undergone by the whole body at each step, which, albeit significantly attenuated, are transmitted to the head [76]. These movements modify the magnetic and capacitive coupling of the user and the electrode leads, leading to an alteration of the parasitic current flowing into the leads [77]. A resulting parasitic voltage drop is then produced in the electrode/gel/skin interface, which interferes with the EEG signal [78]. Finally, electrode movements can also cause impedance variation, which directly affects the electrode voltage offset [79].

Unfortunately, all these *motion artifacts* are not limited to a small spectral band, so they cannot be simply removed by frequency filtering. In a study conducted to assess EEG signal quality in motion environments [80], it is shown that EEG spectra in the walking (or jogging) condition exhibit frequency peaks consistent with the fundamental stride frequency, as well as its harmonics. The authors also state that motion artifacts affect signal integrity most prominently at low frequencies (*i.e.*, <4 Hz) during steady walk. Nevertheless, the study also shows that traditional N1 and P300 event-related potentials (ERP) elicited during a standard *auditory* discrimination task (*i.e.*, “oddball paradigm”) are not dramatically affected by the walking condition, either in amplitude, in topographic distribution or response time (70% of acceptable trials across all participants). This is, however, not the case for the jogging condition, for which only 14% of trials were accepted.

Analog conclusions are drawn in more recent studies, where subjects are standing or walking on a treadmill while performing a *visual* oddball response task [74,81].

#### 3.1.2. Magnetoencephalography

Magnetoencephalography (MEG) detects the weak magnetic fields resulting from the intracellular electrical currents in neurons. The neurophysiological processes that produce MEG signals are the same as those that produce EEG signals. The advantage of MEG is that magnetic fields are less distorted by the skull and scalp than electric fields. This technique offers a spatial resolution of a few millimeters and a temporal resolution of a few milliseconds [82], but requires highly sensitive devices [arrays of SQUIDs (superconducting quantum interference devices)] cooled to a few degrees Kelvin. Additionally, measurements must be realized in a shielded room in order to minimize interferences with magnetic fields from external sources. This non-invasive technique gives only access to shallow parts of the brain and is too bulky and expensive to become an acquisition system suitable for everyday use.

#### 3.1.3. Electrocorticography

Electrocorticography (ECoG) consists of implanting electrodes under the dura mater, directly on the surface of the cortex, without penetrating it. This technique represents a partially invasive compromise, offering a good signal quality and spatial resolution [83]. Compared to EEG, ECoG provides higher temporal and spatial resolution. ECoG signals are characterized by higher amplitudes and a lower vulnerability to artifacts, such as blinks and eye movement [84]. However, this technique is invasive and requires a surgical intervention in order to implant an electrode grid. This operation is thus risky. Early experiments with animals indicated that stable ECoG signals could be recorded over several months [85]. More recent studies with monkeys also indicated that ECoG electrodes remained stable during several months [86]. Nevertheless, the long-term stability of EGoG signals remains unclear to date.

#### 3.1.4. Intracortical Neuron Recording: Brain Implants

Brain implants may be directly inserted into the grey matter of the brain, in order to measure the electrical activity of single neurons. Three types of signals can be obtained with this technology: single-unit activity (SUA), multi-unit activity (MUA) and local field potentials (LFPs) [87]. The SUA is obtained by high-pass filtering (>300 Hz) of the signal of a single neuron. MUA contains the contribution of multiple SUAs. LFPs are computed by low-pass filtering (<300 Hz) of the neuron activity. LFPs are analog signals, whereas SUA and MUA contain the spiking activity of single neurons.

Brain implants provide the best quality of signals, with a much higher spatial and temporal resolution than EEG recording. In 2005, such a type of neurosurgery was done successfully with a tetraplegic, who was subsequently able to move, only by thought, a cursor on a computer screen, as well as an artificial hand [88]. Nevertheless, this technique requires a heavy and risky surgical operation. Additionally, such devices raise several issues, like long-term viability and biocompatibility [89].

#### 3.1.5. Functional Magnetic Resonance Imaging

Functional magnetic resonance imaging (fMRI) is a non-invasive technique allowing one to determine the blood oxygen level variations that occur during brain activity (higher neural activity requires more glucose and oxygen). The main advantage of this technique is a high spatial resolution, of the order of the millimeter, which makes it perfectly suitable for accurately localizing active regions inside the brain [90]. fMRI suffers from a poor time resolution of about one or 2 s. On top of this, this technique is highly susceptible to head motion artifacts. Like for MEG, fMRI requires cumbersome and very expensive equipment, which is not really suited for individual and everyday applications.

#### 3.1.6. Nuclear Functional Imaging Techniques

Single-photon emission-computed tomography (SPECT) is an imaging technique based on the tracking of gamma rays emitted by radionuclides injected in the bloodstream of the patient. Specific chemicals (radioligands), by their particular binding properties to certain types of tissues (e.g., brain tissues), allow one to concentrate the radionuclides in the region of interest of the body, thus making them visible to the gamma cameras of the system [91]. SPECT is a tomography tool that provides 3D information and can reconstruct an image of a thin slice along any chosen axis of the body. The spatial resolution of SPECT is about 1 cm, and several dozens of seconds are needed for a single projection (a full 360° scan by 5° steps takes up to 30 min). When used for functional brain imaging, the system is able to assess the cerebral blood flow, which is directly linked to the local brain metabolism.

Positron-emission tomography (PET) is relatively similar to SPECT. The radionuclides injected in the patient emit positrons, which annihilate with electrons located in the vicinity (a few mm) and, therefore, produce a pair of gamma rays emitted in opposite directions [92]. By detecting the two gammas in coincidence mode, enhanced spatial information is available for the imaging algorithms. Consequently, a better spatial resolution is reached, compared to the SPECT technique. In brain imaging applications, the active molecule generally chosen is FDG, an equivalent of glucose. Again, brain metabolism is assessed in this particular application of the technology.

#### 3.1.7. Near-Infrared Spectroscopy

Near-infrared spectroscopy (NIRS) is another non-invasive acquisition technique. It determines the variations of hemoglobin concentrations linked to neural activity by detecting changes in the optical response (absorption, scattering) of cerebral tissue to near-infrared light. Infrared light penetrates the skull to a depth of approximately 1–3 cm below the surface. Thus, only the outer cortical layer can be imaged using this technique. A further limitation of the technique lies in the fact that hemodynamic response occurs a certain number of seconds after its associated neural activity [93]. The spatial resolution of this technique is of the order of the centimeter, while the time resolution is of approximately 200 ms. Contrary to MEG and fMRI, NIRS is an appropriate measurement modality for everyday use, as its equipment is relatively cheap, portable [94], simple to attach and requires little user training [95].

### 3.2. Spatial Organization of Supra-Spinal Control

Neuroimaging of gait is not straightforward, and practical problems are posed, since the majority of imaging techniques (like PET, fMRI and EEG) require that subjects do not move their head during the experiments. Moreover, functional brain imagery of subjects walking on a treadmill does not allow one to discriminate whether the evoked activity is due to sensory input or motor input. Consequently, alternative neuroimaging techniques have to be employed, like, for instance, recording cerebral activity during motor planning of walking prior to walking initiation, using tasks that share some cerebral processes with gait, without the need to engage in actual gait (like motor imagery of gait or repetitive foot movements). The results obtained using these different strategies are detailed below and summarized in Table 3.

**Table 3:** An overview of the results obtained by different functional neuroimaging studies of gait in healthy subjects.

**Publication**	**Neuroimaging Method**	**Experimental Approach**	**Key Findings**
Fukuyama *et al.*, 1997 [96]	SPECT	Real gait (on ground)	During gait, increased activity in the supplementary motor area (SMA), medial primary sensorimotor area, striatum, cerebellar vermis and visual cortex
Hanakawa *et al.*, 1999 [97]	SPECT	Real gait (on treadmill)	Cerebral activity during walking also observed in the dorsal brainstem
Miyai *et al.*, 2001 [98]	MRS	Real gait (on treadmill)	Walking increases cerebral activity bilaterally in the medial primary sensorimotor cortices and the SMA
Suzuki *et al.*, 2004 [99]	NIRS	Real gait at different speeds (on treadmill)	Increase of cerebral activity in the prefrontal cortex and premotor cortex as locomotor speed increases; cerebral activity in the medial sensorimotor cortex not influenced by locomotor speed
Malouin *et al.*, 2003 [100]	PET	Motor imagery of standing, gait initiation, real walking, walking with obstacles	Motor imagery of walking increases activity in the pre-SMA (compared to imagined standing); in the left visual cortex and caudate nucleus (compared to imagery of gait initiation)
Jahn *et al.*, 2004 [101]	fMRI	Motor imagery of standing, walking and running	Cerebellar activation increased during motor imagery of running, not during motor imagery of walking and standing; vestibular and somatosensory cortex were deactivated during running, but not during walking
Miyai *et al.*, 2001 [98]	NIRS/fMRI	Repetitive foot movements	Foot-extension flexion movements generate a similar brain activation pattern to that associated with walking
Sahyoun *et al.*, 2004 [102]	fMRI	Active *vs.* passive foot movements	During active movements, an increase of cerebral activity in the somatosensory cortex, SMA, cingulate motor area, secondary somatosensory cortex, insular cortices, putamen, thalamus and cerebellum
De Jong *et al.*, 2002 [103]	PET	Antiphase flexion and extension movements	Cerebral activations distributed over the right anterior parietal and right dorsal premotor cortex
Christensen *et al.*, 2000 [104]	PET	Bicycle movements	Both passive and active bicycling increase cerebral activity bilaterally in primary sensorimotor cortices, SMA and the anterior part of the cerebellum.
La Fougère *et a.l.*, 2010 [105]	PET/fMRI	Real *vs.* imagined locomotion	During real and imagined locomotion: activations in the frontal cortex, cerebellum, pontomesencephalic tegmentum, parahippocampal, fusiform and occipital gyri; deactivations in the multisensory vestibular cortices (superior temporal gyrus, inferior parietal lobule). Real steady-state locomotion seems to use a direct pathway via the primary motor cortex, whereas imagined modulatory locomotion uses an indirect pathway via a supplementary motor cortex and basal ganglia loop.

#### 3.2.1. Execution of Real Gait

Despite the experimental difficulties mentioned above, a few techniques allow one to assess cerebral activity during actual gait.

Both SPECT and PET scans, for instance, may be used to study brain activity during actual gait. Indeed, these techniques allow one to separate, in time, task performance from image acquisition. When radioactive substances are injected intravenously during gait, they are rapidly distributed in the brain proportionally to local cerebral blood flow and, most importantly, remain in the brain for hours. Therefore, the spatial distribution of radionuclides at the time of PET or SPECT scanning reflects the pattern of cerebral perfusion at the time of injection. Using this approach, a significant increase in cerebral activity was found during gait in the supplementary motor area (SMA), medial primary sensorimotor area, striatum, cerebellar vermis and visual cortex [96]. This was the first study to show changes in cortical activity during walking in human subjects, compared to the resting state. Later on, the same group demonstrated that a significant cerebral activity during walking is also observed in the dorsal brainstem [97]. This finding is important, because it is one of the few observations suggesting the presence of brainstem locomotor centers in humans.

Cerebral activity can also be monitored while subjects are walking on a treadmill thanks to NIRS. This technique allows the comparison of several experimental conditions. In [106], the cerebral activities evoked during gait, alternating foot movements, arm swing and motor imagery of gait were compared. The results of this study indicated that the gait-related responses along the central sulcus were medial and caudal to the activity associated with arm swing. This is quite in agreement with the known somatotopic organization of the motor cortex. Crucially, these authors showed that walking increased cerebral activity bilaterally in the medial primary sensorimotor cortices and the SMA, and to a greater extent than the alternation of foot movements.

In another NIRS study, the effect of different walking speeds on cerebral activity was examined. It was demonstrated that cerebral activity in the prefrontal cortex and premotor cortex tend to increase as locomotor speed increases, whereas cerebral activity in the medial sensorimotor cortex is not influenced by locomotor speed [99].

#### 3.2.2. Gait Initiation

As already mentioned in this review, EEG recording during walking is particularly challenging, due to motion artifacts. However, some researchers have published EEG studies prior to and/or during gait initiation [107,108]. This experimental approach offers two advantages. First, it provides a high temporal resolution analysis of the electrical brain activity in an action where changes in sensory input are minimal. Second, motion artifacts are drastically reduced, because the recording is realized before the onset of any movement. In these studies, stronger event-related potentials were found in the medial central region (Cz) when comparing EEG activity preceding externally-cued gait initiation with activity preceding foot dorsiflexion. This EEG difference indicates that the medial frontal cortex, above its role in initiating a simple foot movement, supports the initiation of gait [109].

#### 3.2.3. Motor Imagery of Gait

Another strategy to assess the cerebral bases of true gait control consists of investigating motor imagery of gait, *i.e.*, the mental simulation of gait without actual execution. This approach presents the advantage of being totally compatible with techniques like fMRI and PET, which provide relatively high spatial resolution and whole-brain coverage. Numerous studies have been published on the subject. Cerebral activity evoked during motor imagery of standing, initiating gait, walking and walking with obstacles was analyzed in [100]. The authors report that motor imagery of walking increased cerebral activity in the pre-SMA when compared to imagined standing, and in the left visual cortex and caudate nucleus when compared to imagery of gait initiation. Comparing motor imagery of walking with or without obstacles increased cerebral activity in the precuneus bilaterally, the left SMA, the right parietal inferior cortex and the left parahippocampal gyrus. This illustrates that the neuronal circuitry of gait can extend beyond motor cortex, and it can be modulated by the difficulty of the imagined locomotor task.

In another study, based on fMRI, motor imagery of standing, walking and running was studied [101]. The results obtained indicate an increase in the activation of the cerebellum during motor imagery of running, but not during motor imagery of walking and standing. Additionally, vestibular and somatosensory cortex were deactivated during running, but not during walking. As summarized in [109], these findings suggest that the speed of gait is under the control of a cerebellar locomotor center and that cortical processing of vestibular and somatosensory information is particularly important during walking.

#### 3.2.4. Repetitive Leg or Foot Movements

A last approach to assess the supra-spinal control of human locomotion is to study repetitive leg or foot movements. Indeed, it is thought that these movements rely partly on the same neural processes as those used during actual gait. In a combined NIRS and fMRI study, it was shown that foot extension-flexion movements indeed generate a similar brain activation pattern to that associated with walking [98]. Like motor imagery of gait, the study of leg or foot movements presents practical advantages, like the reduction of motion artifacts and the possibility of using cumbersome brain imagery techniques. Of course, one does not study real gait in this case, since this motor task additionally requires the coordination of a large number of body parts and includes the integration of balance control information.

Using fMRI to compare active *vs.* passive unilateral foot extension-flexion movements, it was found that during active compared to passive foot movements, cerebral activity increased in the somatosensory cortex, SMA, cingulate motor area, secondary somatosensory cortex, insular cortices, putamen, thalamus and cerebellum [102]. This suggests that both cortical and subcortical structures are involved in the motor control of rhythmic foot movements.

In a PET study, cerebral activity during antiphase flexion and extension movements of the two upper and the two lower limbs was examined [103]. For both the arms and legs, cerebral activations related to antiphase movements were distributed over the right anterior parietal and right dorsal premotor cortex, suggesting that these structures support the sensorimotor integration required for antiphase movements.

Inter-limb coordination study was assessed with bicycle movements in [104]. The results obtained in this analysis showed that both passive and active bicycling increase cerebral activity bilaterally in primary sensorimotor cortices, SMA and the anterior part of the cerebellum. After subtraction of passive from active bicycling, significant activation was found in the leg area of the primary motor cortex and the precuneus. This suggests that significant cerebral control is involved in the production of rhythmic movements, such as bicycling.

#### 3.2.5. Summary

**Figure 6:**
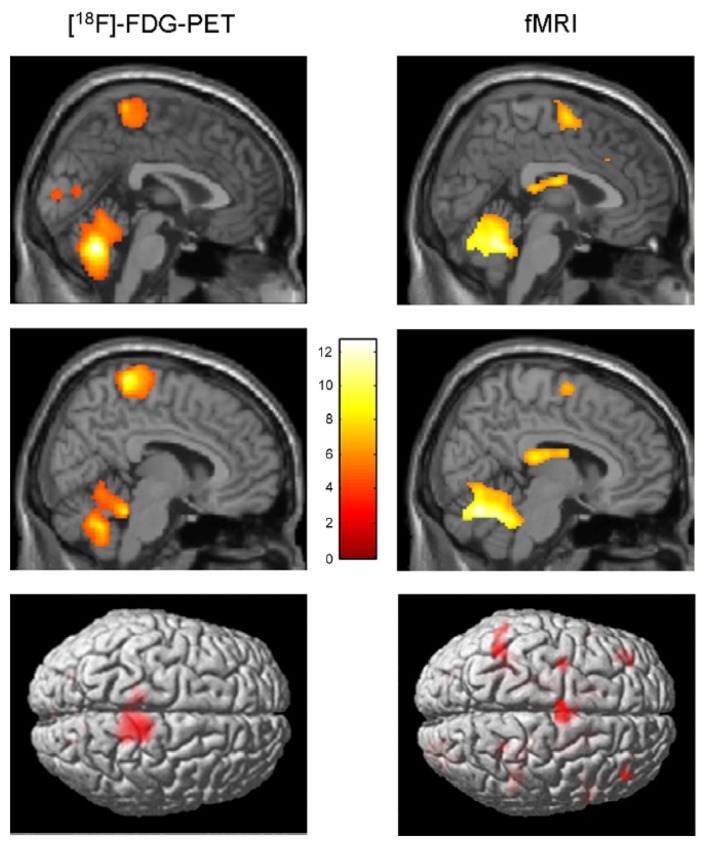
Comparison of real (PET) and imagined locomotion (fMRI) brain activations (this figure is reproduced with permission from [105]; Copyright Elsevier, 2010). Sagittal midline and render views are shown. It can be seen that during real locomotion, the primary motor sensory cortices (pre- and post-central gyri) are active **(left)** as compared to the supplementary motor areas (superior and medial frontal gyri) in mental imagery of locomotion **(right)**. Furthermore during imagined locomotion, the basal ganglia (caudate nucleus, putamen) are active, which is not the case for real locomotion.

Thanks to the numerous neuroimagery studies of gait (or assimilated motor tasks), the supra-spinal control of human locomotion has been identified as lying in different centers in the brainstem, cerebellum and cortex (*cf.* Figure 6). This cerebral network is believed to modulate locomotion (e.g., gait initiation, termination, velocity, direction and spatial orientation) and to control balance and gait by integration of multi-sensory information [110]. The most important regions are the cerebellar locomotor region (CLR), the mesencephalic locomotor region (MLR) and the subthalamic locomotor region (SLR).

Remarkable similarities exist between the real and imagined locomotion networks [105]. The first one is the activation of the midline cerebellar area, which controls body and trunk balance, and the cerebellar locomotor region, which is thought to regulate speed and gives rhythmical impulses to the brainstem and spinal cord [101]. The second remarkable similarity in both paradigms is the activation of occipital visual cortices, which are related to visual processing.

**Figure 7:**
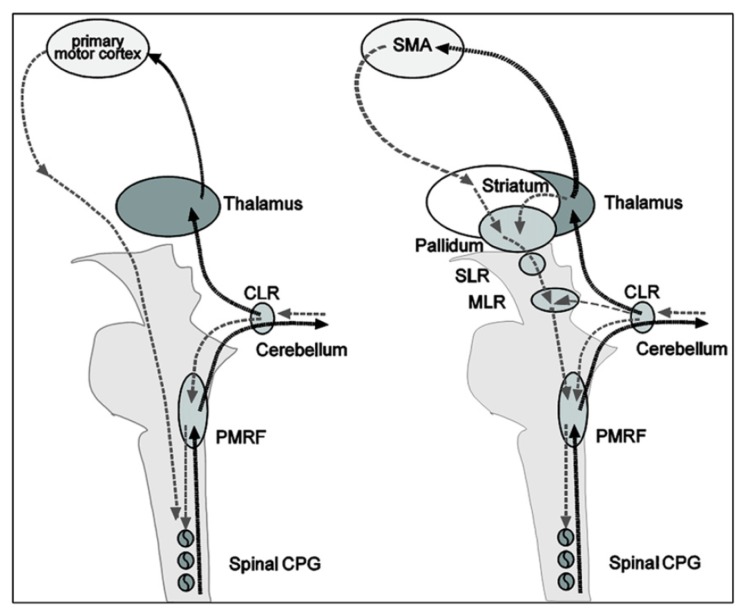
Illustration of the executive and planning networks of locomotion, as suggested in [105]. Execution of locomotion in a non-modulatory steady state **(left side)** goes from the primary motor cortex areas directly to the spinal central pattern generators (CPG), thereby bypassing the basal ganglia and the brainstem locomotor centers. A feedback loop runs from the spinal cord to the cerebellum and, thereby, via the thalamus to the cortex. For planning and modulation of locomotion **(right side)**, cortical locomotor signals originate in the prefrontal supplementary motor areas and are transmitted through the basal ganglia via disinhibition of the subthalamic locomotor region (SLR) and mesencephalic locomotor region (MLR), where they converge with cerebellar signals from the cerebellar locomotor region (CLR). The MLR functionally represents a cross point for motor information form basal ganglia and cerebellar loops. Descending anatomical projections are directed to the medullary and pontine reticular formations (PMRF) and the spinal cord; ascending projections are in the main part concentrated on the basal ganglia and the non-specific nuclei of the thalamus (not shown for the sake of clarity). The CLR also projects via the thalamus back to the cortex. Cortical signals are furthermore modulated via a thalamo-cortical-basal ganglia circuit. The schematic drawing shows a hypothetical concept of a direct pathway of steady-state locomotion **(left)** and an indirect pathway of modulatory locomotion **(right)**. SMA, supplementary motor cortex. This figure is reproduced with permission from [105]; Copyright Elsevier, 2010.

Significant differences between real and imagined locomotion networks have also been found [105]. Whereas the primary motor cortex is activated (in the functional region of the leg) during real locomotion, supplementary motor areas (superior and medial frontal cortex, dorsolateral prefrontal cortex) and basal ganglia (caudate nucleus, putamen) are activated during mental imagery. The most acceptable explanation suggested for this is that the premotor and basal ganglia activations in imagined locomotion could reflect an indirect pathway of locomotion that is responsible for the modulation of locomotion, whereas the primary motor activations in real continuous walking utilize a direct pathway for a steady-state of locomotion (*cf.* Figure 7).

### 3.3. Temporal Characteristics of Supra-Spinal Control

Functional brain imaging techniques have brought a lot of useful information to localize the cerebral centers involved in human locomotion control. The weak point of these techniques, however, reside in the poor temporal resolution they offer. In that regard, electroencephalography (EEG) represents an interesting complementary technique for investigating neural processes governing walk and, particularly, the dynamics of the brain.

Detailed biophysical studies have revealed that single neurons are characterized by a complex dynamics, with the ability to resonate and oscillate at multiple frequencies. Precise timing of their activity within neuronal networks encode information, and synchronous activity of oscillating networks is thought to link the single neuron activity to global behavior [111]. The control of precise actions, like locomotion, for instance, requires the integration of multiple pieces of information and, thus, synchrony of different convergent inputs. One of the roles of oscillatory activities in the brain is to operate this synchrony. Indeed, oscillation-based synchrony is the most energy-efficient physical mechanism for temporal coordination [111]. In that regard, it is fundamental to analyze brain dynamics to understand the mechanisms involved in the supra-spinal centers during locomotion.

However, electrophysiological investigation of the cerebral activity elicited during walking is highly challenging. Indeed, head and body movements constitute an important source of mechanical artifacts strongly affecting the EEG signal quality.

Consequently, the main strategy generally used to overcome these experimental difficulties consists of focusing on simplified foot or leg movements, which imply common cerebral processes with gait. In these experimental protocols, subjects are mainly static and produce only limited lower limb movements. A strong advantage of this approach is, of course, that motion artifacts are drastically limited. In this case, however, the full neural activity related to walk is not available and, for instance, cerebral processes involved in posture and balance control are missing. Recording EEG signals of subjects walking on a treadmill include, of course, all these aspects, but then requires a powerful analysis technique to discriminate the different artifact contributions from the real cortical signal. Analysis results of these two approaches, *static*, on the one hand, and *dynamic*, on the other hand, are reviewed hereinafter.

#### 3.3.1. Electrocortical Potentials Related to Lower Limb Activation in a Static Condition

The cortical activity associated with bilateral anti-phase and in-phase rhythmic foot movements produced by subjects sitting in a chair was investigated in [112]. In this study, the authors found significant corticomuscular coherence between EEG signals and the anterior tibial muscles, at the stepping frequencies in the central midline region, extending further to the frontal mesial area. During isometric co-contraction of the calf muscles, coherence appeared between 15 and 30 Hz, concentrated on the central midline area [Cz-central-parietal (CPz) electrodes]. This is the first study demonstrating that there exists a representation of rhythmic foot motor patterns in the cortex, transmitted to the muscles and fed back to the cortex with delays compatible with fast corticospinal transmission, which may be important for gait control.

Assisted lower limb movements have also been investigated using electroencephalography [113]. In this study, subjects performed standardized, assisted stepping movements (*i.e.*, mimicking walk) in an upright position, while being secured to a tilt table. Electrocortical sources associated with the movement-related potential were localized in the primary motor cortex, the premotor cortex, the supplementary motor cortex, the cingulate cortex, the primary somatosensory cortex and the somatosensory association cortex (*i.e.*, in accordance with the findings of functional brain imagery). The authors demonstrated that a clear succession of activations and deactivations was present in the movement-related potential, in direct relationship with specific phases of the gait-like leg movements. In particular, it was shown that cortical activity was the greatest during transition between flexion and extension of the legs and vice versa.

In [114], a non-invasive EEG-based BCI governing a functional electrical stimulation (FES) system for ankle movement is presented. In this application, healthy subjects perform repetitive foot dorsiflexions. EEG patterns underlying this action are detected in real time, and this information is subsequently used to trigger the FES of the tibialis anterior of the contralateral foot, so as to achieve its dorsiflexion. In fact, the trigger (or non-trigger) information is given by a linear Bayesian classifier trained using a vector of spatio-spectral features, which optimally discriminate the idling and dorsiflexion states. The authors state that analysis of subject-specific prediction models demonstrated that the EEG power changes in the *μ*, *β* and low *γ* bands observed over mid-central areas (*i.e.*, electrode Cz) were the most informative features for classification. This likely corresponds to activity within the primary motor cortex foot representation area and/or supplementary motor area (which is not surprising from a brain anatomy standpoint) and is in perfect agreement with prior studies [115,116].

#### 3.3.2. Electrocortical Potentials Related to Walking

The first analysis of EEG during walking on a treadmill was published by Gwin and co-workers [117]. By using a method based on independent component analysis (ICA) combined with an inverse modeling approach, the authors claimed they could discriminate electrocortical sources, muscle sources and other artifacts from the raw EEG signals. They found that cortical activity in the anterior cingulate, posterior parietal and sensorimotor cortex exhibited significant and smooth intra-stride changes in spectral power. More precisely, alpha and beta band spectral powers increased in or near the left/right sensorimotor and dorsal anterior cingulate cortex at the end of each stance phase (*i. e.*, as the leading foot was contacting the ground and the trailing foot was pushing off). According to this study, power increases in the left/right sensorimotor cortex were more important for contralateral limb push-off (ipsilateral heel strike) than for ipsilateral limb push-off (contralateral heel strike). Finally, the authors reported evidence of intrastride high-gamma spectral power changes in anterior cingulate, posterior parietal and sensorimotor cortex.

In parallel, Presacco and co-workers [118] showed for the first time that the kinematics of the ankle, knee and hip joints during human treadmill walking can be inferred from EEG signals. Successful decoding of these signals was done basically by filtering them (0.1–2 Hz) and passing them through a linear autoregressive model. According to this study, gait trajectories were inferred with accuracies comparable to those from neural decoders based on multiple single-unit activity recorded in non-human primates [1]. The results of this study indicate a high involvement of a fronto-posterior cortical network in the control of walking and suggest that EEG signals can be used to study, in real time, the cortical dynamics of walking and to develop brain-machine interfaces aimed at restoring human gait function.

#### 3.3.3. Spatio-Frequential Characteristics of the Detected Potentials

From the spatial point of view, all the studies found activations of the brain globally compatible with the primary motor cortex's foot representation area and/or supplementary motor area, except one (*cf.* Table 4). Indeed, Presacco and co-workers [118] report the activation of a complex, distributed and sparse cortical network, in which scalp areas over anterior, right lateral and right anterior-occipital scalp areas seem to equally contribute (at least to their decoding of the kinematics of the right leg, for subjects walking on a treadmill). The same results were published in [119].

**Table 4:** EEG studies of human locomotion: a schematic view of recent results obtained with static and dynamic experimental protocols. M1 is the primary motor cortex; PMC is the premotor cortex; SMA is the supplementary motor cortex; CC is the cingulate cortex; S1 is the primary somatosensory cortex; and SA is the somatosensory association cortex; Cz is the medial central region. BCI, brain-computer interface; FES, functional electrical stimulation; ICA, independent component analysis; ERD, event-related synchronization; ERS, event-related desynchronization; CCA, canonical correlation analysis; ERSP, event-related spectral perturbation.

**Publication**	**Aim of the Study**	**Approach/Cleaning Method**	**Activated Brain Areas**	**Frequency Bands of Interest**
Raethjen *et al.*, 2008 [112]	Rhythmic foot movements	Static/no cleaning	Central midline region and frontal mesial area	Stepping frequency and *β* band (15–30 Hz)
Wieser *et al.*, 2010 [113]	Assisted lower-limb movements	Static/no cleaning	Ml, PMC, SMA, CC, S1, SA	No frequency analysis. Activations are directly related to specific phases of the gait-like movements
Do *et al.*, 2011 [114]	BCI dedicated to a FES system for ankle movement	Static/no cleaning	Mid-central areas (electrode Cz)	*μ*, *β* and low-*γ* bands
Gwin *et al.*, 2011 [130]	EEG activity during treadmill walking	Dynamic/ICA cleaning (AMICA)	Anterior cingulate, posterior parietal and sensorimotor cortex	*α* and *β* bands and clear evidence of high-*γ* intra-stride spectral power changes
Presacco *et al.*, 2011 [118]	Neural decoding of treadmill walking from EEG signals	Dynamic/no cleaning	Involvement of a broad fronto-posterior cortical network	Delta band (0.1–2 Hz)
Severens *et al.*, 2012 [125]	Detection of ERD/ERS during walking	Dynamic/CCA cleaning	ERD found in the *μ* band above the electrode Cz and in the *β* band above the lateral motor cortex (electrodes C3 and C4).	ERSPs in *μ* and *β* bands are coupled to the gait cycle with significant differences between left swing, right swing and double support phase of the gait cycle
Wagner *et al.*, 2012 [127]	Robotic-assisted treadmill walking	Dynamic/ICA cleaning (Infomax)	Central midline areas	*μ* and *β* rhythms suppressed during active walking in the Lokomat; modulations of the lower *γ* band (25 to 40 Hz) related to the phases of the gait cycle; these might be related to sensorimotor processing of the lower limbs
Petersen *et al.*, 2012 [129]	Treadmill walking	Dynamic/coherence analysis	Significant coherence between EEG (Cz) and EMG (tibialis anterior) before the heel strike	Coherence between 24 and 40 Hz; evidence that the coupling is not due to non-physiological artifacts

From the frequential point of view, spectral power variations were generally found from alpha to gamma bands, but, astonishingly, a successful neural decoding of treadmill walking was realized by Presacco and co-workers [118] using EEG signals band-pass filtered between 0.1 and 2 Hz. This is particularly surprising, because it was shown in two other studies, conducted to assess EEG signal quality in motion environments [74,80], that EEG spectra in the walking (or jogging) condition exhibit frequency peaks consistent with the fundamental stride frequency, as well as its harmonics. The authors in [80] also state that motion artifacts affect signal integrity most prominently at low frequencies (*i.e.*, the delta band) during steady walk. In their analysis protocol, Presacco and co-workers [118] do not mention any pre-processing method aiming at either correcting or discriminating these motion artifacts from the real cortical signals. The only way for them to make the choice of this frequency band legitimate is the fact that good results are obtained and, moreover, other studies exploited the same portion of the EEG spectrum to decode upper limb movements. We strongly emphasize the fact that, in the latter studies, no motion artifact due to gait is produced. Consequently, this might suggest that the decoding of kinematics of walking (*periodical* movement) on the basis of the EEG signals is done by Presacco and co-workers [118] with a linear autoregressive model exploiting the *periodical* motion artifacts present in the EEG recordings. This option is furthermore supported by the fact that no spectral information is given under 3 Hz in the study of Gwin [120].

This last point is corroborated by a recent paper from Antelis and co-workers [121]. These authors have focused on several publications in which are reported successful reconstructions of different limb kinematics from EEG using the low frequency activity of the EEG and linear regression models [122,123,124]. Antelis and co-workers [121] showed that the mathematical properties of the linear regression model and of the correlation metric used in these studies could explain the good results reported. Moreover, they demonstrated that correlation results obtained with real EEG signals, shuffled or random EEG data were not statistically different. This means that the linear models developed in [122,123,124] are able to provide the same results irrespective of the presence or absence of limb velocity information in EEG signals.

Other recent studies dedicated to EEG analysis during a locomotion task may be added to this discussion. Severens and co-workers [125] investigated the possibility of measuring event-related desynchronizations (ERDs) [126] and event-related spectral perturbations (ERSPs) during walking on a treadmill. After cleaning EMG artifacts using canonical correlation analysis (CCA), they found an ERD in the mu band above the central motor cortex (electrode Cz) and in the beta band above the lateral motor cortex (electrodes C3 and C4). In addition, they found that ERSPs in mu and beta bands were coupled to the gait cycle with significant differences between the left swing, right swing and double support phase of the gait cycle. They did not report any signal of cortical origin at low frequency. Indeed, as the low frequency modulations they found in the ERSPs were also visible in the occipital channels, the authors explained that these were very unlikely related to brain activity and probably due to remaining artifacts.

Wagner and co-workers [127] also showed that mu and beta rhythms are suppressed during active walking in the Lokomat, a robotic gait orthosis. They also provided evidence of modulations of the lower gamma band (25 to 40 Hz), localized in central midline areas and related to the phases of the gait cycle. For different reasons, the authors speculate that these activations and deactivations might be related to sensorimotor processing of the lower limbs in the complex motor pattern of human locomotion. Although their ERSPs plots exhibit ERD and ERS around and below 5 Hz, they neither comment on them nor claim that these originate from cortical activity.

#### 3.3.4. About the Origin of the Detected Signals

Among all the works described in previous paragraph, only [112] tried to determine the origin of the information flux contained in the studied signals (descending commands from the brain or sensory feedback sent to the brain). This is done by computing time delays between EEG time series and electromyographic activity of the involved lower limb muscles by means of the “maximizing coherence method” [128]. Actually, the other studies presented in previous paragraph do not consider this aspect and give no indication on the direction of the brain-muscle interaction (*i.e.*, if it is up-going or down-going). It is therefore unknown, for instance, if the intra-stride spectral power variations found by Gwin[120] are due to voluntary movements or sensory feedback (or a combination of both). The same question arises concerning the EEG decoding presented by Presacco and co-workers [118]. Resolving this ambiguity is particularly crucial, though, for the development of gait rehabilitation systems. Indeed, if the information detected in the EEG signals is purely due to the sensory feedback of the gait-related movements, it would be unusable to drive any device, given that no valid *prediction* of a movement can be done exploiting sensory information *resulting* from it.

Most importantly, studying EEG signals in treadmill walking also requires the need to exclude gait-related artifacts. Too few studies tackle this issue [73]. In particular, [117] used an ICA analysis coupled with an inverse solution approach. These authors claim that they could disentangle muscular contributions and other artifacts from real cortical signals. However, in a previous study, the very same authors [120], using the very same dataset, clearly stated that:

“Unlike more spatially stationary artifacts in EEG signals arising from eye movements, scalp muscles, fMRI gradients, *etc.*, which may be resolved by ICA decomposition into a subspace of one or more independent components, we found that gait-related movement artifact remained in many if not most of the independent components. This prevented us from removing only a small subset of components capturing the movement artifacts.”

For this reason, they considered the removal of motion artifacts from the EEG during walking and running on a treadmill using an artifact template subtraction method. Such a method allowed for enhancing the detection of P300 potentials in ambulatory conditions. Nevertheless, the study of cerebral processes involved in human locomotion is not possible using a subtraction method, as it would undoubtedly remove interesting signals from the EEG recordings. For this reason, the authors used only the ICA approach to clean the EEG signals [117]. In this study, the issue of motion artifacts was completely eluded, and no mention was made of any appropriate treatment to reject them. Thus, it can be doubted that the time-frequency analysis plots shown in that paper do not contain any motion artifact contribution. In the discussion conducted in [73], it was shown that a time-frequency analysis of the signal of an accelerometer placed on the head of a subject walking at 1.67 m/s on a treadmill presented periodic power spectral changes over large frequency bands, in a similar way to the results obtained after ICA by [117].

Finally, it should be noted that on the basis of a spectral analysis, it is not possible to determine which cortical region is directly involved in the transmission of motor commands to the muscles. In contrast, coherence analysis reveals anatomical coupling between cortical activity and the motor output to the muscles by detecting common rhythmicities in EMG and EEG signals. In the study conducted in [129], the coupling between electroencephalographic (EEG) and electromyographic (EMG) signals from leg muscles during treadmill walking was investigated. The authors report significant coherence between EEG signals recorded over the leg motor area (Cz electrode) and EMG from the tibialis anterior (TA) muscle in the 24–40 Hz frequency band before the heel strike, during the swing phase of the gait cycle. The presence of a significant imaginary part of the complex coherence indicates that the coupling in the study was not due to non-physiological artifacts. The negative sign of this imaginary part of the coherence suggests that the cortical activity was leading the muscle activity. Time lag estimates between EEG and EMG signals are consistent with the typical cortico-spinal conduction times. This result indicates that rhythmic cortical activity in this particular frequency band is transmitted to the lower limb muscles during walking, at specific moments in the gait cycle. This work thus proves and confirms that the motor cortex directly contributes to the muscle activity involved in human locomotion. On top of this, according to the significant coherence values (24–40 Hz around Cz) found by [129], the multiple ERD-ERS detected by Gwin [130] in the 3–24 and 40–76 Hz bands are obviously not indicative of a direct corticospinal drive, at least, not to the tibialis anterior. Thus, one may think that these signals, if not affected by residual artifacts, would rather reflect the control of sensory afferents (*i.e.*, one of the hypotheses formulated by the authors in [130] themselves). It is interesting to note that the studies by Wagner [127] and Severens [125] do not report multiple ERD-ERS in the *α*, *β* and *γ* bands and are in line with the coherence study made by [129].

### 3.4. Results from Invasive Studies

Although this paper is essentially devoted to non-invasive analyses of human locomotion, it may be interesting to report a few important results from invasive studies in order to bring supplementary information to the different elements presented above.

Several invasive studies with mammals report rhythmic cerebral activations in phase with the gait cycle. As mentioned in [131], olivary neurons, in rats, discharge rhythmically at frequencies closely matching the step cycle [132]. Analogously, in cats, the locomotion activity of more than 90% of neurons of motor cortex are modulated in the rhythm of strides [133]. Moreover, it was shown that the discharge rate means, peaks and depths of stride-related frequency modulation changed dramatically during accurate stepping, as compared with simple walking [134].

In a recent breakthrough [1], bipedal walking patterns could be extracted from the modulations of discharge rates of monkey primary somatosensory cortex (S1) and primary motor cortex (M1) neuronal ensembles. In this paper, the activity modulations in hundreds of simultaneously recorded neurons were analyzed, and it was demonstrated that both M1 and S1 neurons modulated their firing rate in relation to the gait cycle. Remarkably, the firing rate of each neuron peaked at a particular phase of the stepping cycle. Using a set of linear decoders (Wiener filters), the authors could thus predict locomotion kinematic parameters with a very satisfying accuracy. Large neuronal ensembles were needed for accurate predictions of leg kinematics, and the number of units required increased with the task complexity. Furthermore, the authors report a superior performance of neuronal populations drawn from several cortical areas in predicting movement kinematics compared to the performance of populations drawn from a single area. Moreover, results indicated that both M1 and S1 neurons contributed significantly to the prediction of the leg kinematics. As expected, M1 modulations were more useful for predicting future values in the parameters of walking, whereas S1 modulations better predicted the past values. This observation, however, must still be confirmed with more experiments, since accurate predictions of future values of locomotion parameters could be obtained from S1 activity in one of the two monkeys participating in the study. Nevertheless, this work provides the first proof of concept that, in the future, real-time neuroprosthetic systems for restoring bipedal walking in severely paralyzed patients could be implemented.

Interesting results were also obtained with electrocorticography. Significant coherence between right sensorimotor cortex and distal left leg muscles was found up to 60 Hz during voluntary induced myoclonic jerks. Additional higher frequency coherence (∼140 and 190 Hz) was found during sensory-induced myoclonic jerks [135]. Recently, significant decreases (4–7, 8–14 and 15–25 Hz) or increases (26–45 and 65–95 Hz) in power (compared to the rest) were reported during spontaneous movement of the hand and/or arm contralateral to electrode grid placement [136]. Furthermore, specific high gamma ECoG responses were also identified during natural expressive speech and natural motor tasks involving upper and lower extremities [137]. Thanks to these particular features, several research teams have demonstrated that prosthesis control based on ECoG signals is quite feasible [138,139,140,141]. Nevertheless, these promising advances have been made for upper limb applications. It remains to be shown if the same principles can be successfully exploited for developing walk rehabilitation systems.

**Figure 8:**
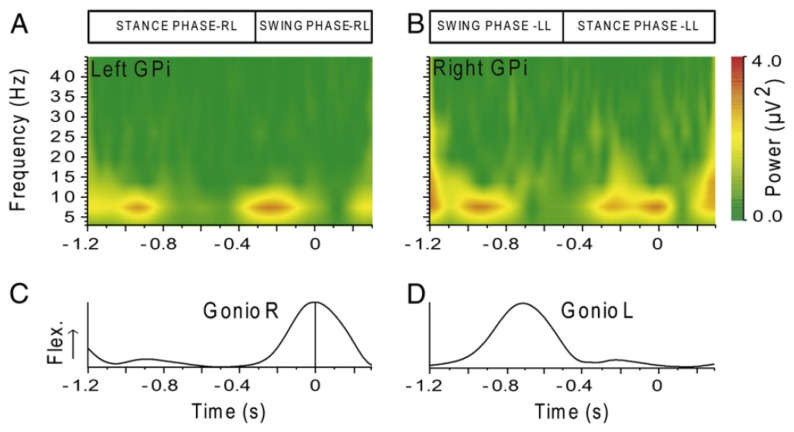
Time-frequency plots (wavelet transformation) of LFP oscillations during gait cycle. Upper row (**A**) and (**B**): analyzed electrode pair. The right electrode pair is on the right side. (**C**) and (**D**): goniometer traces. Modulation of LFPs occurs in the 6–11 Hz frequency range. In this frequency band, amplitudes are upregulated during the early stance phase and swing phase of the contralateral leg. LL: left leg; RL: right leg; Gonio: goniometer; Flex: flexion. This figure is reproduced with permission from [142]; Copyright Elsevier, 2011.

The last original invasive study that is worth mentioning in this review is the first analysis of basal ganglia activity recorded from deep brain stimulation (DBS) electrodes in human subjects during treadmill walking [142]. Recordings were made with patients who have DBS electrodes located in the globus pallidus internum (GPi) for treatment of neck and upper trunk motor impairments, with no gait disturbances. The authors report that local field potentials (LFP) spectra of GPi recordings made during walking showed significantly higher power values in the lower frequency bands (4–12 Hz) and in the gamma band (60–90 Hz) as compared to during sitting or standing. The opposite was seen in the beta band (15–25 Hz), where the power was significantly reduced during walking. According to the authors, these changes may initiate or sustain gait-related activity in locomotor brainstem centers. No significant differences over all frequency bands were observed between the sitting and the standing conditions. Additionally, a modulation of the amplitudes in the theta-alpha (6-11 Hz) range was seen in all subjects. The maximum amplitude variation was located between 6 and 11 Hz during the early stance phase of the left leg in the right hemisphere and symmetrically on the left during the early stance phase of the right leg (*cf.* Figure 8). This modulation seems to indicate that information about individual gait cycles is also present in the basal ganglia.

## 4. Development of Non-Invasive Brain-Computer Interfaces Dedicated to Rehabilitation Systems

After reviewing the main principles of human locomotion control and, in particular, the spatial and temporal characteristics of the supra-spinal control, the next paragraphs will deal with the development of rehabilitation systems based on non-invasive brain-computer interfaces (BCI). After defining the concept of BCI, different challenges, like detecting the movement intention based on EEG signals or translating EEG signals to valuable commands dedicated to rehabilitation systems, will be reviewed and discussed.

### 4.1. General Considerations about BCIs

**Figure 9:**
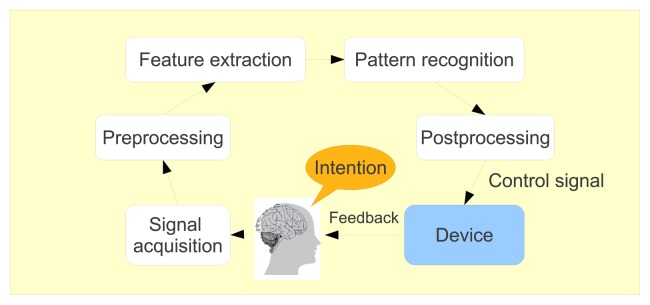
General scheme of a classical brain-computer interface (BCI): first of all, the subject performs a specific mental task in order to produce a signal of interest in his brain; then, this signal is acquired and generally pre-processed in order to get rid of different artifacts. Afterwards, some discriminating features are extracted and classified (pattern recognition) to determine which specific signal was produced. Finally, the identified signal is associated with a specific action to be performed by a computer or any electronic device.

Brain-computer interfaces (BCI) include devices or systems that respond to neural or cognitive processes. These systems enable their users, whose neural system may have been destroyed by amputation, trauma or disease, to control a computer or any robotic device by interpreting neurophysiological signals, which are recorded and processed following different steps, as shown in Figure 9. First, the brain signals are pre-processed to clean them as much as possible. Then, some features are extracted and classified so that the computer can determine in which mental state the user was. Finally, the corresponding action is produced by the system. As it is non-invasive, light and relatively cheap, electroencephalography (EEG) is the most used acquisition technique to record cerebral activity of the BCI users.

Thanks to current BCI technology, severely disabled people can communicate [143], control computers [144] or drive robotic [145] or simple prosthetic devices [146] via the power of their brain only, without activating any muscles. Nowadays, BCI applications dedicated to both disabled and healthy users are also being developed in the video game field [147]. Although functional, BCI technology offers information transfer rates that are too limited to control complex systems entirely. Consequently, shared control is used extensively in assistive applications. This means that the BCI user generally sends high-level commands to the system, which is able to operate all the low-level problems [1,148]. Very interestingly, the benefits of BCI are not limited to the control of simple electric devices. It has been shown that the simple fact of learning to operate a BCI has a positive impact on brain plasticity, with a significant increase in motor cortical excitability and a modification of the brain network topology [149]. As such, BCI could become a viable tool for new post-stroke rehabilitation strategies [150].

In the case of BCI dedicated to walk rehabilitation systems, the first challenge is to detect the user intention (to start walking, to stop, to go faster, to slow down, to turn left or right, *etc.*). Then, the system has to generate a realistic human walking movement, corresponding to the detected user intent. Finally, a feedback should be sent to the user, to help him control the system. These are the main challenges to raise. They are detailed in the following sections, with an emphasis on existing systems and the latest results obtained.

### 4.2. Detecting the User Intent

Detecting the user intent, and, particularly, predicting the next movement onset, type and direction, is of first interest, as it could activate rehabilitation devices in a fully natural way. Although very few gait intent detection studies have been undertaken [151,152], predicting the leg movement is of primary importance and would definitely be helpful to adequately control an orthosis/prosthesis. Indeed, from a mechanical point of view, the only interest of using brain signals to control a prosthesis would be to detect early willingness for the next move [153]. Thereby, the whole device could anticipate the movement and adjust the mechanical system as precisely as possible. It has to be underlined that this anticipation is not feasible solely on the basis of physical sensors placed on the rehabilitation system, since these detect the movement only once it has been produced.

**Figure 10:**
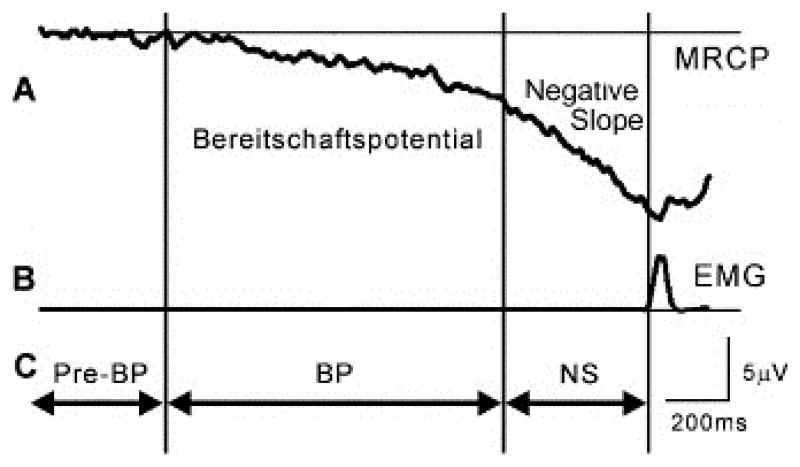
When looking at a Bereitschaftspotential (BP) signal, three main sections are observed: no potential, a slow decreasing potential early BP and a steeper late BP (this figure is reproduced with permission from [154]; Copyright Elsevier, 2005). MRCP is the movement-related cortical potential.

**Figure 11:**
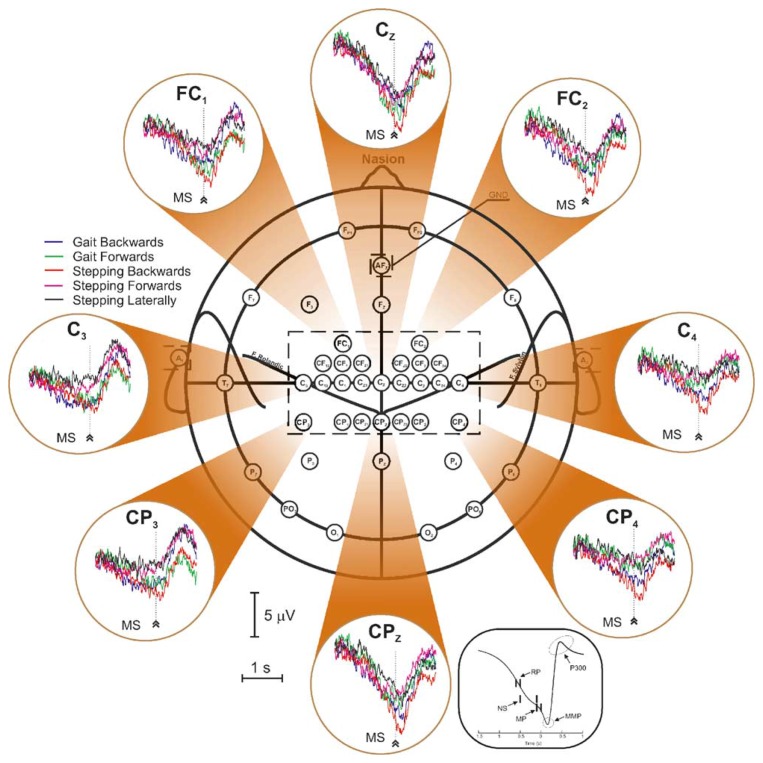
It clearly appears that the BP potentials are similar for all five tasks. A classification between those tasks would be difficult. The potentials are strong over the motor cortex area close to the midline (MS is the movement start). This figure is reproduced with permission from [108]; Copyright Elsevier, 2005.

From a practical point of view, researchers have basically two options to detect the movement intention thanks to EEG signals: look for the emergence of a Bereitschaftspotential (BP) or detect the production of an event-related (de-)synchronization (ERD/ERS) by the brain. The BP, on the one hand, is a slow cortical potential that deepens in negativity about 1.5 s to 1 s before the movement onset. As shown in Figure 10, the BP potential can be divided into three different parts. First, in the pre-BP section, the brain signals are not affected by the movement intent yet. Then, in the BP section, or *early BP*, the potential slowly decreases about 1.5 s before movement onset and is more prominent in the central-medial areas. Second, the negative slope (NS), or *late BP*, corresponds to a steeper slope and starts around 400 ms prior to movement onset. This potential is mainly localized over the primary motor cortex (M1). It was shown that BP potentials are also observed in the brain just before the imagination of movements [155]. In a recent study [108], the BP signal was analyzed during gait intent. Five different tasks were performed: gait/stepping forward, gait and stepping backward and stepping laterally. As shown in Figure 11, the measured potentials vary slightly, depending on the experimental paradigm. The most important potentials and the most notable variations occur at the top of the head, around electrode Cz, which corresponds to the leg representation in the motor and sensorimotor cortices.

The event-related desynchronization, on the other hand, manifests itself as a short-lasting decrease of power in some specific frequency bands [151]. This decrease can start as early as 2 s before movement onset. Generally, the ERDs occur in the *μ* (8–12 Hz) and *β* (12–30 Hz) bands, which are directly linked to movement planning and execution. These frequency limits, as well as the strength and laterality of the ERD patterns depend on the subject, especially if the patient suffers from stroke impairment [156].

As reviewed in [151], several researchers have succeeded in automatically detecting BP or ERD, in a non-invasive way. While most of the studies focus on the upper limb (finger, wrist, arm), only three studies tackle the detection of ankle dorsiflexion movement without cues [114,157]. The obtained true positive rates range between 82.5% [157] and 100% [114]. Another study evaluated the possibility to classify right-hand, left-hand, tongue or right-foot movements with less success [158]. Very recently, the single-trial classification of gait and point movement preparation was investigated [152]. Depending on the analyzed pair of tasks, the average error rate was 25%.

Finally, motor-imagery was also investigated as an effective BCI paradigm to detect the intention to start walking. It was shown in [159] that paraplegic and tetraplegic patients could trigger a walking simulator by imagining themselves walking or idling. In these two experimental conditions, the EEG power in the 9–13 Hz band in the mid-frontal (FCz), central (Cz) and central-parietal (CPz) areas contained the best discriminant information. Based on these features, classification results estimated offline ranged from about 60% to 90%. This proves that SCI patients have the possibility to operate a robust BCI walking simulator with a short training period and satisfying accuracy. This expands the results of a similar analysis that had been undertaken earlier [145]. All these studies are summarized in Table 5.

**Table 5:** Several studies of EEG signals preceding lower limb motor tasks are available. The presented results show that it should be feasible to activate a prosthetic/rehabilitation device. SCI, spinal cord injury.

**Publication**	**Acquisition Protocol**	**Brain Feature**	**Key Findings**
Niazi *et al.*,2011 [157]	Ankle dorsiflexion movement without cues	BP	Predicted the movement with an average true positive rate of 82.5% around 187 ms before movement onset
Morash *et al.*, 2008 [158]	Perform or imagine right-hand, left-hand, tongue or right-foot movement after a “Go” cue.	ERD/ERS	Predicting which of the four movements/imageries is about to occur is possible (around 40% accuracy)
Velu *et al.*, 2013 [152]	Natural walking from a starting position to a designated ending position, pointing at a designated position from the starting position or remaining standing at the starting position.	BP	Significant classification achieved for all conditions, with errors for movement *vs.* standing around 25% when averaged across nine subjects
Do *et at.*, 2011 [114] Do *et at.*, 2012 [160]	Detection of EEG patterns related to repetitive foot dorsiflexions	Activations in the Cz electrode	Foot lifter rehabilitation system based on FES (online performance near 100% using FFT-based features)
King *et al.*, 2013 [159]	Motor imagery of walking or idling executed by participants with paraplegia or tetraplegia due to SCI	ERD/ERS	The EEG power in the 9–13 Hz band in the mid-frontal (FCz), central (Cz) and central-parietal (CPz) areas were the most different, comparing walking and idling imagery; classification accuracy from 60 to 90%
Leeb *et al.*, 2007 [145]	One tetraplegic patient imagines movements of his paralyzed feet	Detection of *β* bursts during imagery	Control of a wheelchair in a virtual environment (go/stop). Classification results ranging from 90 to 100%

### 4.3. Developing a Direct Brain Signal to Limb Kinematics Decoding

Once the movement intent is detected, the rehabilitation system should produce realistic movements of the lower limbs. Ideally, these movements should be produced according to the patient's needs and/or wishes, in order to render the locomotion process almost instinctive, like in real walking. To this end, direct translation of the brain signals into limb kinematics is desirable. Such results, as previously mentioned, have been obtained with monkeys on the basis of invasive experiments, in the context of bipedal walking [1], direct cortical control of 3D neuroprosthetic devices [161] and cortical control of a prosthetic arm for self-feeding [162]. Several studies analyze this approach as summarized in Table 6.

**Table 6:** Only one study suggests a direct decoding of brain signals into lower limb kinematics. However, it was severely called into question by [121].

**Publication**	**BCI Paradigm**	**Rehabilitation Device or Technique**	**Remarks**
Presacco *et al.*, 2011 [118]	EEG to kinematics translation	None	Tries to reproduce results obtained by invasive studies (the RMSvalue between prediction and measurement is around 0.68); highly criticized by [121]
Wagner *et al.*, 2012 [127]	EEG	Lokomat	No control: evaluation of level of participation in robotic-assisted treadmill walking (Fz and Pz difference between active and passive walk)
Tanaka *et al.*, 2013 [163]	NIRS	Exoskeleton	Body motion support-type mobile suit Comparison of the cerebral activity during walking using the suit and normal gait without the suit; recommendations: most effective for gait training to actually walk and not stay fixed in one location; important for patients to swing their arms during gait training in rehabilitation

An attempt to reproduce these results with non-invasive signals (EEG) has been made with human subjects [118], as reported earlier in this review, but seems to be unfruitful, according to the arguments developed in [121]. In the same underlying philosophy, robotic-assisted gait analysis has shown a difference between active and passive walking around the Pz and Cz electrodes [127]. By automatically detecting these modifications, a monitoring of the brain activation could be performed and determine the best rehabilitation procedure, which could help one to recover more quickly. For instance, after analyzing normal gait with and without a support exoskeleton in [163], the authors suggest that efficient lower limb rehabilitation should be based on actual walking and not staying fixed in one location. Additionally, they reported that it is important for patients to swing their arms during gait training in rehabilitation. Anyway, future investigations will be needed to improve the procedure and verify, after all, if the recorded signals contain, or not, sufficient information to control a prosthetic/rehabilitation device permanently. In the meanwhile, other strategies have been developed to achieve a similar goal.

### 4.4. Producing a Gait/Stepping Movement in a Step by Step Approach

The alternative strategy to direct translation of EEG signals into walk kinematics signals consists of detecting high level commands from EEG (like accelerate, decelerate, turn left, right, *etc.*), in order to subsequently produce the desired movement using a dedicated algorithm [164,165]. Generally, this algorithm will drive a functional electrical stimulation (FES) system, a prosthesis, orthosis or exoskeleton [166,167]. To detect high level commands from brain signals, several BCI paradigms can be exploited using visual stimuli in order to elicit P300 potentials or steady-state visually evoked potentials (SSVEPs) (see [65] for a review on BCI). Tactile stimulation could also be exploited to this aim [168].

To convert high level commands into kinematics, a class of algorithms called programmable central pattern generators (PCPGs) is particularly suited for rehabilitation systems dedicated to walking. Inspired by biological CPG, the PCPG algorithm is able to learn practically any periodical pattern (a gait pattern, for instance) and automatically reproduce it [169]. Very advantageously, the amplitude and frequency of the patterns produced may be changed on the fly, without producing any abrupt change in the output command signal sent to the exoskeleton actuators. By analyzing the gait parameters of healthy subjects walking on a treadmill, our research group showed that adequately combining the amplitude and frequency of the PCPG allowed us to produce realistic gait kinematic patterns in a range of walking speeds from 1.5 to 6 km/h [164].

Actually, only a few studies were performed under an ambulatory context. After a pilot study investigating the feasibility of mobile auditory P300 detection [170], it was more precisely shown that the P300 potential is not affected that much while walking [74,81,120]. Thus, although slow, this approach obtained strongly reliable performance showing the feasibility of the link of a BCI with an external foot lifter orthosis [171]. Based on a SSVEP paradigm, a preliminary study scanning the brain response from 10 Hz to 46 Hz has shown that walking is not a transparent process [172]. In another study using two flickering stimuli, it was shown that ICA and canonical correlation analysis (CCA) were able to extract an SSVEP response while walking [173].

When considering studies in the context of lower-limb rehabilitation, some other papers are available. Using an SSVEP paradigm, it was shown that a lower limb exoskeleton could be controlled by both a step by step approach and in a continuous way [174]. In [175], a walk rehabilitation system that recognizes five types of intention is proposed and an 80% accuracy is claimed. In [176], motor imagery-based BCI allows the subject to make a humanoid robot turn right, turn left and walk forward. The most important results are summarized in Table 7.

**Table 7:** A few applications. SSVEP, steady-state visually evoked potential; PCPG, programmable central pattern generator.

**Publication**	**BCI Paradigm**	**Rehabilitation Device or Technique**	**Remarks**
Cheron *et.al.*, 2012 [165]	EEG (BCI)	Exoskeleton (LOPES)/foot lifter orthosis	Results from the Mindwalker and Bio-fact projects
Mc Daid *et.al.*, 2013 [174]	EEC (SSVEP)	Control of a lower limb exoskeleton; studies motion intent detection and continuous control by the BCI	The control appears feasible
Duvinage *et at.*, 2013 [172]	EEG (SSVEP)	Scanning of the brain response from 10 Hz to 46 Hz	Gait influences the SSVEP brain response
Duvinage *et at.*, 2012 [171]	EEG (P300) plus PCPG	Foot lifter orthosis	Reliable proof of concept
Li *et al.*, 2012 [176]	Humanoid robot control thanks to BCI	None	Identification of mental activities when the subject is thinking “turning right,” “turning left” or “walking forward”
Zhang *et al.*, 2011 [175]	EEG (SSVEP) plus CPG	None	Walk rehabilitation system that recognizes five types of intention related to human walking; successful classification accuracy above 80%; functional using online EEG data

## 5. Discussion and Considerations for Future Work

Recently, several studies dedicated to rehabilitation purposes have emerged in the non-invasive BCI field. It appears that investigations for upper limbs are more often realized than for lower limbs. This is probably due to both the inherent experimental difficulty of measuring EEG signals in the ambulatory context and the challenging goal of balance control in walk rehabilitation tasks. Another reason is that walking may be considered an automatic movement merely based on reflexes governed at the spinal level. However, it has been recently confirmed that the motor cortex is particularly active during specific phases of the gait cycle, particularly before the foot comes in contact with the ground [177]. Tremendous progress in understanding human locomotion control has been made thanks to invasive studies and neuroimaging techniques, giving the opportunity to build a bridge from BCI to new rehabilitation systems and strategies.

Indeed, traditional approaches aiming at walk recovery after stroke, for instance, may be considered as bottom-up approaches: they focus on the physical level (bottom) in order to influence the neural system (top), being able to rehabilitate the patients because of the mechanisms of neural plasticity. How these mechanisms are established is not well understood. Recently, some authors have promoted the top-down approach, which consists of defining rehabilitation therapies based on the state of the brain after stroke [178]. Mental simulation of movement engages the primary motor cortex in a similar way that motor execution does. This mental exercise, called motor imagery (MI), induces distinctive modulations of sensorimotor rhythms, which can be detected online. A specific BCI technology based on such an MI paradigm can thus be used to help patients in cognitively rehearsing their physical skills in a safe, repetitive manner, even in the case of no residual motor function [178]. This is an example in which BCIs are extensively exploited to promote neuroplasticity in combination with traditional physiotherapy and robot-aided therapy.

Nevertheless, before developing fully efficient neuronal prosthesis control systems, several challenging problems remain to be solved. These have already been mentioned throughout this review. They are summarized below.

According to the recent results presented in the previous sections, it seems quite possible to successfully detect the intention of walking on the basis of EEG signals only (go/no-go detection) [151]. However, determining the precise orientation of the movement by analyzing the shape of the Bereitschaftspotential happens to be much more challenging, given the weak difference between the BP associated with forward and backward movements [108].

Successful conversion of brain signals into limb kinematics has been achieved with monkeys, on the basis of invasive measurements [1]. Recently, an attempt to realize a similar conversion with EEG signals in humans has been made [118], but the statistical significance of the obtained results remains to be clearly established [121]. In particular, the question arises if the effective bit rate that can be achieved with EEG technology is sufficient to constantly drive a robotic arm or leg.

By contrast, non-invasive EEG signals can be effectively exploited to run standard brain-computer interfaces. These BCI can be designed to decode high level orders (like, for instance, “go faster”, “go slower”, “turn left”, “stop”, *etc.*), while adapted algorithms, such as PCPG, provide the rehabilitation system actuators with meaningful limb kinematics [164,165]. It was shown in several publications that standard BCI paradigm performances are not much affected by the numerous artifacts polluting EEG signals recorded under “gentle” ambulatory conditions [74,170]. The use of external visual stimuli (with P300 or SSVEP BCI paradigms) may, however, be considered cumbersome and too tiring by patients.

In order to develop better BCIs for walk rehabilitation, fundamental research aiming at detecting the precise role of motor cortex during the gait cycle (and other movements, like stair climbing) should be pursued. It is indeed important to figure out what can effectively and non-ambiguously be measured using EEG: descending commands from the motor cortex; integration of ascending sensorimotor information; artifacts (probably a mix of these different contributions, but in which proportions?). In this context, it seems necessary to define several experimental protocols in order to disentangle the different signals.

To this end, we propose characterizing the descending brain commands that are involved in human walk control in a *static* approach (inspired by [112]), in order to ensure the absence of EEG motion artifacts. To this aim, the EEG signals of subjects sitting on a chair would be recorded. The subjects would then be asked to produce voluntary rhythmic foot movements, staying at the same tempo. The feet will not be in contact with the ground, to ensure a minimal sensorimotor feedback. Several tempos would be produced. Furthermore, EEG should be recorded when the subject is sitting and not moving the feet, to define a baseline, necessary when using brain imagery tools, like LORETA[179]. To assess the presence (or absence) of motion artifacts, an accelerometer should be placed on the neck. A complete characterization of these data could then be realized, by analyzing the event-related spectral perturbations (ERSP) combined with a time-warping transformation [117], by computing directed corticomuscular coherence and, in particular, delays between EEG and EMG time series (to assess the information flow direction). Then, characterization of EEG signals caused by somatosensory information coming from the feet of the subject when sitting (again, to prevent any motion artifacts) should be undertaken. More precisely, the same experiment as above could be realized, with the feet in contact with ground, this time. By comparing the two states (contact/no contact), it would be possible to emphasize the contribution of sensory feedback. Alternatively, one could use special tactors to stimulate the feet, mimicking the sensation of walk and study the properties of the EEG signals that are phase-locked with this stimulation. Finally, if motion artifacts are correctly rejected, provided we know the signals due to descending commands (voluntary rhythmic movements) and those due to tactile stimulation (tactors, mimicking the sensation of walking), we should be able to disentangle the contribution of posture and balance control when the subject is standing and walking.

Directed coherence (Granger causality) is a promising way to disentangle descending from ascending contributions in EEG signals. Unlike coherence, which measures correlation and, therefore, does not allow one to identify the direction of interaction between two signals, Granger causality can provide information about possible causal relationships. Using such an analysis, directed coherence in both the descending (EEG to EMG) and ascending (EMG to EEG) directions was found in beta frequencies, in human subjects performing a precision grip task [180]. This study provides, for the first time, clear evidence of bidirectional corticomuscular coherence in man. Such an analysis should also be made in the context of bipedal walking.

Of course, another crucial research axis to further develop deals with the adequate cleaning of motion artifacts in EEG signals. The first available option consists of mathematically correcting corrupted signals. The most popular method is independent component analysis (ICA), which consists of separating EEG into subcomponents (independent components) in such a way that these are all statistically independent of each other. By discarding components related to artifacts, it is possible to reconstruct cleaned EEG signals [69]. While this technique is really powerful to eliminate basic artifacts (like eye motion or blink artifacts), it seems not so well adapted to motion artifacts produced during walking, since these cannot be completely separated from cortical signals, as explained in [120]. Furthermore, the weak point of this technique resides in the arbitrary nature of the choice of independent components to eliminate. Therefore, improved cleaning methods are desirable, especially if the goal is to directly convert EEG signals into lower limb kinematics. On the other hand, it has been demonstrated that motion artifacts do not dramatically impact low complexity P300-based BCI under ambulatory conditions [74], for instance. Interestingly, it has been shown that artifact-resistant measures could be computed in order to detect cognitive EEG activity during locomotion [119]. This may constitute a useful perspective for the future. Finally, one could also mention the idea of developing a device able to determine the motion artifacts corrupting the EEG electrode online, in order to correct them with adaptive filtering by optimal projection [181].

From a fundamental point of view, we would like to underline the fact that the mechanism of gait control may change as a function of the walking speed, since the kinematics and EMG patterns during the gait cycle vary significantly in shape under 3 km/h [17]. Thus, it should be interesting to systematically compare EEG signals during walking at very low, normal and high speeds.

To conclude, it is really exciting to see that new BCI applications are being developed in the field of rehabilitation. These realizations are based on the considerable knowledge acquired over time in the field of brain sciences. In this review, we have summarized the main principles of human locomotion control, described the first non-invasive BCIs dedicated to walk rehabilitation systems and identified the main technical challenges ahead in the field. We must also not forget that the patients should always be put at the heart of the development process [182], by integrating their personal needs and preferences, in order to produce the best possible benefit for their rehabilitation.

## References

[1] Fitzsimmons N., Lebedev M., Peikon I., Nicolelis M.A. (2009). Extracting kinematic parameters for monkey bipedal walking from cortical neuronal ensemble activity. Front. Integr. Neurosci..

[2] Courtine G., Gerasimenko Y., van den Brand R., Yew A., Musienko P., Zhong H., Song B., Ao Y., Ichiyama R., Lavrov I. (2009). Transformation of nonfunctional spinal circuits into functional states after the loss of brain input. Nat. Neurosci..

[3] Harkema S., Gerasimenko Y., Hodes J., Burdick J., Angeli C., Chen Y., Ferreira C., Willhite A., Rejc E., Grossman R.V.E. (2011). Effect of epidural stimulation of the lumbosacral spinal cord on voluntary movement, standing, and assisted stepping after motor complete paraplegia: A case study. Lancet.

[4] Cheron G., Bengoetxea A., Bouillot E., Lacquaniti F., Dan B. (2001). Early emergence of temporal co-ordination of lower limb segments elevation angles in human locomotion. Neurosci. Lett..

[5] Cheron G., Bouillot E., Dan B., Bengoetxea A., Draye J., Lacquaniti F. (2001). Development of a kinematic coordination pattern in toddler locomotion: Planar covariation. Exp. Brain Res..

[6] Nielsen J.B. (2003). How we walk: Central control of muscle activity during human walking. Neuroscientist.

[7] Ivanenko Y., Dominici N., Cappellini G., Dan B., Cheron G., Lacquaniti F. (2004). Development of pendulum mechanism and kinematic coordination from the first unsupported steps in toddlers. J. Exp. Biol..

[8] Bédard S. (2004). Control System and Method for Controlling an Actuated Prosthesis.

[9] Ragnarsdottir H.G., Clausen A.V., Jonsson H. (2007). Control System and Method for Controlling an Actuated Prosthesis.

[10] Moser D., Ewins D.J. (2006). A Control System for a Lower Limb Prosthesis or Orthosis.

[11] Goffer A. (2002). Gait-Locomotor Apparatus.

[12] Nandi G.C., Ijspeert A.J., Nandi A. Biologically Inspired CPG Based Above Knee Active Prosthesis.

[13] Sankai Y. Leading Edge of Cybernics: Robot Suit HAL.

[14] Ferris D., Lewis C. Robotic Lower Limb Exoskeletons Using Proportional Myoelectric Control.

[15] Hargrove L., Huang H., Schultz A., Lock B., Lipschutz R., Kuiken T. Toward the Development of a Neural Interface for Lower Limb Prosthesis Control.

[16] Uustal H., Baerga E. (2004). Physical Medicine and Rehabilitation Board Review.

[17] Van Hedel H., Dietz V. (2010). Rehabilitation of locomotion after spinal cord injury. Restor. Neurol. Neurosci..

[18] Kandel E., Schwartz J., Jessell T. (2000). Principles of Neural Science.

[19] Grillner S., Wallen P. (1985). Central pattern generators for locomotion, with special reference to vertebrates. Annu. Rev. Neurosci..

[20] Duysens J., de Crommert H.W.V. (1998). Neural control of locomotion; Part 1: The central pattern generator from cats to humans. Gait Posture.

[21] Dimitrijevic M., Gerasimenko Y., Pinter M. (1998). Evidence for a spinal central pattern generator in humans. Ann. N. Y. Acad. Sci..

[22] Yang J.F., Gorassini M. (2006). Spinal and brain control of human walking: Implications for retraining of walking. Neuroscientist.

[23] Thomas A., Autgaerden S., Education, S.S.M.; Unit I (1966). Locomotion from Pre- to Post-Natal Life: How the Newborn Begins to Acquire Psycho-Sensory Functions.

[24] De Vries J., Visser G., Prechtl H. (1982). The emergence of fetal behaviour. I. Qualitative aspects. Early Hum. Dev..

[25] Ivanenko Y.P., Dominici N., Cappellini G., Di Paolo A., Giannini C., Poppele R.E., Lacquaniti F. (2013). Changes in the spinal segmental motor output for stepping during development from infant to adult. J. Neurosci..

[26] Robinson S.R., Smotherman W.P. (1992). Fundamental motor patterns of the mammalian fetus. J. Neurobiol..

[27] Westerga J., Gramsbergen A. (1993). Development of locomotion in the rat: The significance of early movements. Early Hum. Dev..

[28] Ho S.M. (1997). Rhythmic motor activity and interlimb co-ordination in the developing pouch young of a wallaby (*Macropus eugenii*). J. Physiol..

[29] Yang J.F., Stephens M.J., Vishram R. (1998). Transient disturbances to one limb produce coordinated, bilateral responses during infant stepping. J. Neurophysiol..

[30] Dominici N., Ivanenko Y.P., Cappellini G., d'Avella A., Mond V., Cicchese M., Fabiano A., Silei T., Di Paolo A., Giannini C. (2011). Locomotor primitives in newborn babies and their development. Science.

[31] Holmes G. (1915). The goulstonian lectures on spinal injuries of warfare. Br. Med. J..

[32] Kuhn R.A. (1950). Functional capacity of the isolated human spinal cord. Brain.

[33] Calancie B., Needham-Shropshire B., Jacobs P., Willer K., Zych G., Green B.A. (1994). Involuntary stepping after chronic spinal cord injury: Evidence for a central rhythm generator for locomotion in man. Brain.

[34] Coleman R.M., Pollak C.P., Weitzman E.D. (1980). Periodic movements in sleep (nocturnal myoclonus): Relation to sleep disorders. Ann. Neurol..

[35] Lugaresi E., Cirignotta F., Coccagna G., Montagna P. (1986). Nocturnal myoclonus and restless legs syndrome. Adv. Neurol..

[36] Shik M., Orlovskii G., Severin F. (1968). Locomotion of the mesencephalic cat elicited by stimulation of the pyramids. Biophysics.

[37] Gerasimenko I., Makarovski A., Nikitin O. (2000). Control of the human and animal locomotor activity in the absence of supraspinal effects. Ross. Fiziol. Zh. Im. I. M. Sechenova.

[38] Gerasimenko Y., Avelev V., Nikitin O., Lavrov I. (2003). Initiation of locomotor activity in spinal cats by epidural stimulation of the spinal cord. Neurosci. Behav. Physiol..

[39] Dorofeev I., Avelev V., Shcherbakova N., Gerasimenko Y. (2008). The role of cutaneous afferents in controlling locomotion evoked by epidural stimulation of the spinal cord in decerebrate cats. Neurosci. Behav. Physiol..

[40] Duysens J., Clarac F., Cruse H. (2000). Load-regulating mechanisms in gait and posture: Comparative aspects. Physiol. Rev..

[41] Pang M.Y.C., Yang J.F. (2000). The initiation of the swing phase in human infant stepping: Importance of hip position and leg loading. J. Physiol..

[42] De Crommert H.W.V., Mulder T., Duysens J. (1998). Neural control of locomotion: Sensory control of the central pattern generator and its relation to treadmill training. Gait Posture.

[43] Schomburg E.D., Petersen N., Barajon I., Hultborn H. (1998). Flexor reflex afferents reset the step cycle during fictive locomotion in the cat. Exp. Brain Res..

[44] Pearson K.G. (1995). Proprioceptive regulation of locomotion. Curr. Opin. Neurobiol..

[45] Zehr E.P., Duysens J. (2004). Regulation of arm and leg movement during human locomotion. Neuroscientist.

[46] Christensen L.O.D., Morita H., Petersen N., Nielsen J. (1999). Evidence suggesting that a transcortical reflex pathway contributes to cutaneous reflexes in the tibialis anterior muscle during walking in man. Exp. Brain Res..

[47] Geyer H., Herr H. (2010). A muscle-reflex model that encodes principles of legged mechanics produces human walking dynamics and muscle activities. IEEE Trans. Neural Syst. Rehabil. Eng..

[48] Whelan P.J. (1996). Control of locomotion in the decerebrate cat. Prog. Neurobiol..

[49] Jordan L.M., Pratt C.A., Menzies J.E. (1979). Locomotion evoked by brain stem stimulation: Occurrence without phasic segmental afferent input. Brain Res..

[50] Eidelberg E., Walden J., Nguyen L. (1981). Locomotor control in macaque monkeys. Brain.

[51] Barbeau H., Rossignol S. (1987). Recovery of locomotion after chronic spinalization in the adult cat. Brain Res..

[52] Pearson K.G., Rossignol S. (1991). Fictive motor patterns in chronic spinal cats. J. Neurophysiol..

[53] Barbeau H., Chau C., Rossignol S. (1993). Noradrenergic agonists and locomotor training affect locomotor recovery after cord transection in adult cats. Brain Res. Bull..

[54] Rossignol S., Barbeau H. (1993). Pharmacology of locomotion: An account of studies in spinal cats and spinal cord injured subjects. J. Am. Paraplegia Soc..

[55] Forssberg H., Grillner S. (1973). The locomotion of the acute spinal cat injected with clonidine I.V. Brain Res..

[56] Petersen N.T., Butler J.E., Marchand-Pauvert V., Fisher R., Ledebt A., Pyndt H.S., Hansen N.L., Nielsen J.B. (2001). Suppression of EMG activity by transcranial magnetic stimulation in human subjects during walking. J. Physiol..

[57] Grillner S., Walln P., Saitoh K., Kozlov A., Robertson B. (2008). Neural bases of goal-directed locomotion in vertebrates—An overview. Brain Res. Rev..

[58] Dobkin B.H., Firestine A., West M., Saremi K., Woods R. (2004). Ankle dorsiflexion as an fMRI paradigm to assay motor control for walking during rehabilitation. NeuroImage.

[59] Harada T., Miyai I., Suzuki M., Kubota K. (2009). Gait capacity affects cortical activation patterns related to speed control in the elderly. Exp. Brain Res..

[60] Halliday D.M., Conway B.A., Christensen L., Hansen N.L., Petersen N.P., Nielsen J.B. (2003). Functional coupling of motor units is modulated during walking in human subjects. J. Neurophysiol..

[61] Capaday C. (2002). The special nature of human walking and its neural control. Trends Neurosci..

[62] Nielsen J.B. (2002). Motoneuronal drive during human walking. Brain Res. Rev..

[63] Paul C., Bellotti M., Jezernik S., Curt A. (2005). Development of a human neuro-musculo-skeletal model for investigation of spinal cord injury. Biol. Cybern..

[64] Buxton R.B., Uluda K., Dubowitz D.J., Liu T.T. (2004). Modeling the hemodynamic response to brain activation. NeuroImage.

[65] Nicolas-Alonso L., Gomez-Gil J. (2012). Brain computer interfaces—A review. Sensors.

[66] Baillet S., Mosher J., Leahy R. (2001). Electromagnetic brain mapping. IEEE Signal Process. Mag..

[67] Nunez P., Srinivasan R., Westdorp A., Wijesinghe R., Tucker D., Silberstein R., Cadusch P. (1997). EEG coherency I: Statistics, reference electrode, volume conduction, Laplacians, cortical imaging, and interpretation at multiple scales. Electroencephalogr. Clin. Neurophysiol..

[68] Fisch B., Spehlmann R. (1999). Fisch and Spehlmann's EEG Primer: Basic Principles of Digital and Analog EEG.

[69] Delorme A., Makeig S. (2004). EEGLAB: An open source toolbox for analysis of single-trial EEG dynamics including independent component analysis. J. Neurosci. Methods.

[70] Campos Viola F., Thorne J., Edmonds B., Schneider T., Eichele T., Debener S. (2009). Semi-automatic identification of independent components representing EEG artifact. Clin. Neurophysiol..

[71] Ferdousy R., Choudhory A., Islam M., Rab M., Chowdhory M. Electrooculographic and electromyographic artifacts removal from EEG.

[72] Nolan H., Whelan R., Reilly R. (2010). FASTER: Fully automated statistical thresholding for EEG artifact rejection. J. Neurosci. Methods.

[73] Castermans T., Duvinage M., Cheron G., Dutoit T. EEG and Human Locomotion-Descending Commands and Sensory Feedback should be Disentangled from Artifacts Thanks to New Experimental Protocols Position Paper.

[74] Castermans T., Duvinage M., Petieau M., Hoellinger T., Saedeleer C., Seetharaman K., Bengoetxea A., Cheron G., Dutoit T. (2011). Optimizing the performances of a P300-based brain-computer interface in ambulatory conditions. IEEE J. Emerg. Sel. Top. Circ. Syst..

[75] Thompson T., Steffert T., Ros T., Leach J., Gruzelier J. (2008). EEG applications for sport and performance. Methods.

[76] Ratcliffe R., Holt K. (1997). Low frequency shock absorption in human walking. Gait Posture.

[77] Misulis K., Head T. (2003). Essentials of Clinical Neurophysiology.

[78] Vanrijn A., Peper A., Grimbergen C. (1990). High-quality recording of bioelectric events. Part 1. Interference reduction, theory and practice. Med. Biol. Eng. Comput..

[79] De Talhouet H., Webster J. (1996). The origin of skin-stretch-caused motion artifacts under electrodes. Physiol. Meas..

[80] Kerick S.E., Oie K.S., McDowell K. (2009). Assessment of EEG Signal Quality in Motion Environments.

[81] Gramann K., Gwin J., Bigdely-Shamlo N., Ferris D., Makeig S. (2010). Visual evoked responses during standing and walking. Front. Hum. Neurosci..

[82] Barnes G.R., Hillebrand A., Fawcett I.P., Singh K.D. (2004). Realistic spatial sampling for MEG beamformer images. Hum. Brain Mapp..

[83] Krusienski D., Grosse-Wentrup M., Galán F., Coyle D., Miller K., Forney E., Anderson C. (2011). Critical issues in state-of-the-art brain-computer interface signal processing. J. Neural Eng..

[84] Ball T., Kern M., Mutschler I., Aertsen A., Schulze-Bonhage A. (2009). Signal quality of simultaneously recorded invasive and non-invasive EEG. NeuroImage.

[85] Yuen T.G., Agnew W.F., Bullara L.A. (1987). Tissue response to potential neuroprosthetic materials implanted subdurally. Biomaterials.

[86] Chao Z.C., Nagasaka Y., Fujii N. (2010). Long-term asynchronous decoding of arm motion using electrocorticographic signals in monkeys. Front. Neuroeng..

[87] Waldert S., Pistohl T., Braun C., Ball T., Aertsen A., Mehring C. (2009). A review on directional information in neural signals for brain-machine interfaces. J. Physiol. Paris.

[88] Abbott A. (2006). Neuroprosthetics: In search of the sixth sense. Nature.

[89] Grand L., Wittner L., Herwik S., Göthelid E., Ruther P., Oscarsson S., Neves H., Dombovári B., Csercsa R., Karmos G. (2010). Short and long term biocompatibility of NeuroProbes silicon probes. J. Neurosci. Methods.

[90] De Charms R., Christoff K., Glover G.H., Pauly J.M., Whitfield S., Gabrieli J.D. (2004). Learned regulation of spatially localized brain activation using real-time fMRI. NeuroImage.

[91] Jaszczak R., Coleman R. (1985). Single photon emission computed tomography (SPECT): Principles and instrumentation. Investig. Radiol..

[92] Phelps M.E., Hoffman E.J., Mullani N.A., Ter-Pogossian M.M. (1975). Application of annihilation coincidence detection to transaxial reconstruction tomography. J. Nuclear Med..

[93] Coyle S.M., Ward T.E., Markham C.M. (2007). Brain-computer interface using a simplified functional near-infrared spectroscopy system. J. Neural Eng..

[94] Vaithianathan T., Tullis I., Everdell N., Leung T., Meek J., Delpy D. (2003). Functional imaging of the brain using a portable NIR instrument. Proc. SPIE Int. Soc. Opt. Eng..

[95] Coyle S., Ward T., Markham C., McDarby G. (2004). On the suitability of near-infrared (NIR) systems for next-generation brain-computer interfaces. Physiol. Meas..

[96] Fukuyama H., Ouchi Y., Matsuzaki S., Nagahama Y., Yamauchi H., Ogawa M., Kimura J., Shibasaki H. (1997). Brain functional activity during gait in normal subjects: A SPECT study. Neurosci. Lett..

[97] Hanakawa T., Katsumi Y., Fukuyama H., Honda M., Hayashi T., Kimura J., Shibasaki H. (1999). Mechanisms underlying gait disturbance in Parkinson's disease: A single photon emission computed tomography study. Brain.

[98] Miyai I., Tanabe H.C., Sase I., Eda H., Oda I., Konishi I., Tsunazawa Y., Suzuki T., Yanagida T., Kubota K. (2001). Cortical mapping of gait in humans: A near-infrared spectroscopic topography study. NeuroImage.

[99] Suzuki M., Miyai I., Ono T., Oda I., Konishi I., Kochiyama T., Kubota K. (2004). Prefrontal and premotor cortices are involved in adapting walking and running speed on the treadmill: An optical imaging study. NeuroImage.

[100] Malouin F., Richards C.L., Jackson P.L., Dumas F., Doyon J. (2003). Brain activations during motor imagery of locomotor-related tasks: A PET study. Hum. Brain Mapp..

[101] Jahn K., Deutschlnder A., Stephan T., Strupp M., Wiesmann M., Brandt T. (2004). Brain activation patterns during imagined stance and locomotion in functional magnetic resonance imaging. NeuroImage.

[102] Sahyoun C., Floyer-Lea A., Johansen-Berg H., Matthews P. (2004). Towards an understanding of gait control: Brain activation during the anticipation, preparation and execution of foot movements. NeuroImage.

[103] De Jong B., Leenders K., Paans A. (2002). Right parieto-premotor activation related to limb-independent antiphase movement. Cereb. Cortex.

[104] Christensen L., Johannsen P., Sinkjaer T., Petersen N., Pyndt H., Nielsen J. (2000). Cerebral activation during bicycle movements in man. Exp. Brain Res..

[105] La Fougère C., Zwergal A., Rominger A., Förster S., Fesl G., Dieterich M., Brandt T., Strupp M., Bartenstein P., Jahn K. (2010). Real versus imagined locomotion: A [18F]-FDG PET-fMRI comparison. NeuroImage.

[106] Miyai I., Tanabe H., Sase I., Eda H., Oda I., Konishi I., Tsunazawa Y., Suzuki T., Yanagida T., Kubota K. (2001). Cortical mapping of gait in humans: A near-infrared spectroscopic topography study. NeuroImage.

[107] Yazawa S., Shibasaki H., Ikeda A., Terada K., Nagamine T., Honda M. (1997). Cortical mechanism underlying externally cued gait initiation studied by contingent negative variation. Electroencephalogr. Clin. Neurophysiol..

[108] Do Nascimento O.F., Nielsen K.D., Voigt M. (2005). Influence of directional orientations during gait initiation and stepping on movement-related cortical potentials. Behav. Brain Res..

[109] Bakker M., Verstappen C.C.P., Bloem B.R., Toni I. (2007). Recent advances in functional neuroimaging of gait. J. Neural Transm..

[110] Rossignol S., Dubuc R., Gossard J.P. (2006). Dynamic sensorimotor interactions in locomotion. Physiol. Rev..

[111] Buzski G., Draguhn A. (2004). Neuronal oscillations in cortical networks. Science.

[112] Raethjen J., Govindan R., Binder S., Zeuner K.E., Deuschl G., Stolze H. (2008). Cortical representation of rhythmic foot movements. Brain Res..

[113] Wieser M., Haefeli J., Bütler L., Jäncke L., Riener R., Koeneke S. (2010). Temporal and spatial patterns of cortical activation during assisted lower limb movement. Exp. Brain Res..

[114] Do A.H., Wang P.T., King C.E., Abiri A., Nenadic Z. (2011). Brain computer interface controlled functional electrical stimulation system for ankle movement. J. NeuroEng. Rehabil..

[115] Neuper C., Pfurtscheller G. (1996). Post-movement synchronization of beta rhythms in the EEG over the cortical foot area in man. Neurosci. Lett..

[116] Solis-Escalante T., Mller-Putz G., Pfurtscheller G. (2008). Overt foot movement detection in one single Laplacian EEG derivation. J. Neurosci. Methods.

[117] Gwin J., Gramann K., Makeig S., Ferris D. (2011). Electrocortical activity is coupled to gait cycle phase during treadmill walking. NeuroImage.

[118] Presacco A., Goodman R., Forrester L., Contreras-Vidal J. (2011). Neural decoding of treadmill walking from non-invasive, electroencephalographic (EEG) signals. J. Neurophysiol..

[119] Lau T., Gwin J., McDowell K., Ferris D. (2012). Weighted phase lag index stability as an artifact resistant measure to detect cognitive EEG activity during locomotion. J. NeuroEng. Rehabil..

[120] Gwin J., Gramann K., Makeig S., Ferris D. (2010). Removal of movement artifact from high-density EEG recorded during walking and running. J. Neurophysiol..

[121] Antelis J.M., Montesano L., Ramos-Murguialday A., Birbaumer N., Minguez J. (2013). On the usage of linear regression models to reconstruct limb kinematics from low frequency EEG signals. PLoS One.

[122] Agashe H., Contreras-Vidal J. Reconstructing Hand Kinematics during Reach to Grasp Movements from Electroencephalographic Signals.

[123] Bradberry T.J., Gentili R.J., Contreras-Vidal J.L. (2010). Reconstructing three-dimensional hand movements from noninvasive electroencephalographic signals. J. Neurosci..

[124] Bradberry T.J., Gentili R.J., Contreras-Vidal J.L. (2011). Fast attainment of computer cursor control with noninvasively acquired brain signals. J. Neural Eng..

[125] Severens M., Nienhuis B., Desain P., Duysens J. Feasibility of Measuring Event Related Desynchronization with Electroencephalography during Walking.

[126] Pfurtscheller G., da Silva F.L. (1999). Event-related EEG/MEG synchronization and desynchronization: Basic principles. Clin. Neurophysiol..

[127] Wagner J., Solis-Escalante T., Grieshofer P., Neuper C., Müller-Putz G., Scherer R. (2012). Level of participation in robotic-assisted treadmill walking modulates midline sensorimotor EEG rhythms in able-bodied subjects. NeuroImage.

[128] Govindan R., Raethjen J., Kopper F., Claussen J., Deuschl G. (2005). Estimation of time delay by coherence analysis. Phys. A Stat. Mech. Appl..

[129] Petersen T.H., Willerslev-Olsen M., Conway B.A., Nielsen J.B. (2012). The motor cortex drives the muscles during walking in human subjects. J. Physiol..

[130] Gwin J.T., Gramann K., Makeig S., Ferris D.P. (2011). Electrocortical activity is coupled to gait cycle phase during treadmill walking. NeuroImage.

[131] Iosa M., Fusco A., Marchetti F., Morone G., Caltagirone C., Paolucci S., Peppe A. (2013). The golden ratio of gait harmony: Repetitive proportions of repetitive gait phases. BioMed Res. Int..

[132] Smith S. (1997). Step cycle-related oscillatory properties of inferior olivary neurons recorded in ensembles. Neuroscience.

[133] Marlinski V., Nilaweera W.U., Zelenin P.V., Sirota M.G., Beloozerova I.N. (2012). Signals from the ventrolateral thalamus to the motor cortex during locomotion. J. Neurophysiol..

[134] Beloozerova I.N., Farrell B.J., Sirota M.G., Prilutsky B.I. (2010). Differences in movement mechanics, electromyographic, and motor cortex activity between accurate and nonaccurate stepping. J. Neurophysiol..

[135] Marsden J., Ashby P., Rothwell J., Brown P. (2000). Phase relationships between cortical and muscle oscillations in cortical myoclonus: Electrocorticographic assessment in a single case. Clin. Neurophysiol..

[136] Vansteensel M., Bleichner M., Dintzner L., Aarnoutse E., Leijten F., Hermes D., Ramsey N. (2013). Task-free electrocorticography frequency mapping of the motor cortex. Clin. Neurophysiol..

[137] Ruescher J., Iljina O., Altenmüller D.M., Aertsen A., Schulze-Bonhage A., Ball T. (2013). Somatotopic mapping of natural upper- and lower-extremity movements and speech production with high gamma electrocorticography. NeuroImage.

[138] Yanagisawa T., Hirata M., Saitoh Y., Goto T., Kishima H., Fukuma R., Yokoi H., Kamitani Y., Yoshimine T. (2011). Real-time control of a prosthetic hand using human electrocorticography signals. J. Neurosurg..

[139] Shin D., Watanabe H., Kambara H., Nambu A., Isa T., Nishimura Y., Koike Y. (2012). Prediction of muscle activities from electrocorticograms in primary motor cortex of primates. PLoS One.

[140] Benz H., Zhang H., Bezerianos A., Acharya S., Crone N., Zheng X., Thakor N. (2012). Connectivity analysis as a novel approach to motor decoding for prosthesis control. IEEE Trans. Neural Syst. Rehabil. Eng..

[141] Pistohl T., Schmidt T.S.B., Ball T., Schulze-Bonhage A., Aertsen A., Mehring C. (2013). Grasp detection from human ECoG during natural reach-to-grasp movements. PLoS One.

[142] Singh A., Kammermeier S., Plate A., Mehrkens J.H., Ilmberger J., Btzel K. (2011). Pattern of local field potential activity in the globus pallidus internum of dystonic patients during walking on a treadmill. Exp. Neurol..

[143] Farwell L., Donchin E. (1988). Talking off the top of your head: Toward a mental prosthesis utilizing event-related brain potentials. Electroencephalogr. Clin. Neurophysiol..

[144] Wolpaw J.R., Birbaumer N., McFarland D.J., Pfurtscheller G., Vaughan T.M. (2002). Brain-computer interfaces for communication and control. Clin. Neurophysiol..

[145] Leeb R., Friedman D., Müller-Putz G.R., Scherer R., Slater M., Pfurtscheller G. (2007). Self-paced (Asynchronous) BCI control of a wheelchair in virtual environments: A case study with a tetraplegic. Comput. Intell. Neurosci..

[146] Pfurtscheller G., Müller G.R., Pfurtscheller J., Gerner H.J., Rupp R. (2003). ‘Thought’—Control of functional electrical stimulation to restore hand grasp in a patient with tetraplegia. Neurosci. Lett..

[147] Lécuyer A., Lotte F., Reilly R.B., Leeb R., Hirose M., Slater M. (2008). Brain-computer interfaces, virtual reality, and videogames. Computer.

[148] Del R. Millán J., Rupp R., Mueller-Putz G., Murray-Smith R., Giugliemma C., Tangermann M., Vidaurre C., Cincotti F., Kubler A., Leeb R. (2010). Combining brain-computer interfaces and assistive technologies: State-of-the-art and challenges. Front. Neurosci..

[149] Pichiorri F., Fallani F.D.V., Cincotti F., Babiloni F., Molinari M., Kleih S.C., Neuper C., Kbler A., Mattia D. (2011). Sensorimotor rhythm-based braincomputer interface training: The impact on motor cortical responsiveness. J. Neural Eng..

[150] Fallani F.D.V., Pichiorri F., Morone G., Molinari M., Babiloni F., Cincotti F., Mattia D. (2013). Multiscale topological properties of functional brain networks during motor imagery after stroke. NeuroImage.

[151] Ahmadian P., Cagnoni S., Ascari L. (2013). How capable is non-invasive EEG data of predicting the next movement? A mini review. Front. Hum. Neurosci..

[152] Velu P., de Sa V.R. (2013). Single-trial classification of gait and point movement preparation from human EEG. Front. Neurosci..

[153] Duvinage M., Jimenez-Fabian R., Castermans T., Verlinden O., Dutoit T. An Active Foot Lifter Orthosis Based on a PCPG Algorithm.

[154] Wasaka T., Nakata H., Kida T., Kakigi R. (2005). Gating of SEPs by contraction of the contralateral homologous muscle during the preparatory period of self-initiated plantar flexion. Cogn. Brain Res..

[155] Lucas M.F., Doncarli C., Farina D., do Nascimento O. Optimization of a Set of Wavelets for Classification of Imaginary Movement-Related Cortical Potentials.

[156] Kaiser V., Daly I., Pichiorri F., Mattia D., Mller-Putz G.R., Neuper C. (2012). Relationship between electrical brain responses to motor imagery and motor impairment in stroke. Stroke.

[157] Niazi I.K., Jiang N., Tiberghien O., Nielsen J.F., Dremstrup K., Farina D. (2011). Detection of movement intention from single-trial movement-related cortical potentials. J. Neural Eng..

[158] Morash V., Bai O., Furlani S., Lin P., Hallett M. (2008). Classifying EEG signals preceding right hand, left hand, tongue, and right foot movements and motor imageries. Clin. Neurophysiol..

[159] King C., Wang P., Chui L., Do A., Nenadic Z. (2013). Operation of a brain-computer interface walking simulator for individuals with spinal cord injury. J. NeuroEng. Rehabil..

[160] Do A., Wang P., King C., Schombs A., Cramer S., Nenadic Z. Brain-Computer Interface Controlled Functional Electrical Stimulation Device for Foot Drop Due to Stroke.

[161] Taylor D.M., Tillery S.I.H., Schwartz A.B. (2002). Direct cortical control of 3D neuroprosthetic devices. Science.

[162] Velliste M., Perel S., Spalding M.C., Whitford A.S., Schwartz A.B. (2008). Cortical control of a prosthetic arm for self-feeding. Nature.

[163] Tanaka E., Saegusa S., Yuge L. (2013). Development of a whole body motion support type mobile suit and evaluation of cerebral activity corresponding to the cortical motor areas. J. Adv. Mech. Des. Syst. Manuf..

[164] Duvinage M., Castermans T., Hoellinger T., Cheron G., Dutoit T. Modeling Human Walk by PCPG for Lower Limb Neuroprosthesis Control.

[165] Cheron G., Duvinage M., de Saedeleer C., Castermans T., Bengoetxea A., Petieau M., Seetharaman K., Hoellinger T., Dan B., Dutoit T. (2012). From spinal central pattern generators to cortical network: Integrated BCI for walking rehabilitation. Neural Plast..

[166] Cowan R., Fregly B., Boninger M., Chan L., Rodgers M., Reinkensmeyer D. (2012). Recent trends in assistive technology for mobility. J. NeuroEng. Rehabil..

[167] Dollar A., Herr H. (2008). Lower extremity exoskeletons and active orthoses: Challenges and state-of-the-art. IEEE Trans. Robot..

[168] Brouwer A.M., van Erp J.B.F. (2010). A tactile P300 brain-computer interface. Front. Neurosci..

[169] Ijspeert A.J. (2008). Central pattern generators for locomotion control in animals and robots: A review. Neural Netw..

[170] Lotte F., Fujisawa J., Touyama H., Ito R., Hirose M., Lécuyer A. (2009). Towards Ambulatory Brain-Computer Interfaces: A Pilot Study With P300 Signals.

[171] Duvinage M., Castermans T., Jimenez-Fabian R., Hoellinger T., de Saedeleer C., Petieau M., Seetharaman K., Cheron G., Verlinden O., Dutoit T. A Five-State P300-Based Foot Lifter Orthosis: Proof of Concept.

[172] Duvinage M., Castermans T., Petieau M., Hoellinger T., Seetharaman K., Cheron G., Dutoit T. A PreliminaryFundamental Study of Ambulatory SSVEP.

[173] Lin Y.P., Wang Y., Jung T.P. A Mobile SSVEP-based Brain-Computer Interface For Freely Moving Humans; The Robustness of Canonical Correlation Analysis to Motion Artifacts.

[174] McDaid A., Xing S., Xie S. Brain Controlled Robotic Exoskeleton for Neurorehabilitation.

[175] Zhang D., Yao L., Wang Y., Zhu X. Functional Interface Between Brain and Central Pattern Generator for Application in Human-Machine System.

[176] Li W., Jaramillo C., Li Y. Development of Mind Control System for Humanoid Robot through a Brain Computer Interface.

[177] Castermans T., Duvinage M. (2013). Corticomuscular coherence revealed during treadmill walking: Further evidence of supraspinal control in human locomotion. J. Physiol..

[178] Belda-Lois J.M., Mena-del Horno S., Bermejo-Bosch I., Moreno J., Pons J., Farina D., Iosa M., Molinari M., Tamburella F., Ramos A. (2011). Rehabilitation of gait after stroke: A review towards a top-down approach. J. NeuroEng. Rehabil..

[179] Pascual-Marqui R. (2002). Standardized low-resolution brain electromagnetic tomography (sLORETA): Technical details. Methods Find. Exp. Clin. Pharmacol..

[180] Witham C.L., Riddle C.N., Baker M.R., Baker S.N. (2011). Contributions of descending and ascending pathways to corticomuscular coherence in humans. J. Physiol..

[181] Boudet S., Peyrodie L., Forzy G., Pinti A., Toumi H., Gallois P. (2012). Improvements of adaptive filtering by optimal projection to filter different artifact types on long duration EEG recordings. Comput. Methods Programs Biomed..

[182] Duvinage M., Castermans T., Petieau M., Seetharaman K., Hoellinger T., Cheron G., Dutoit T. A Subjective Assessment of a P300 BCI System for Lower-Limb Rehabilitation Purposes.

